# Neither measurement error nor speed–accuracy trade-offs explain the difficulty of establishing attentional control as a psychometric construct: Evidence from a latent-variable analysis using diffusion modeling

**DOI:** 10.3758/s13423-025-02696-4

**Published:** 2025-07-14

**Authors:** Alodie Rey-Mermet, Henrik Singmann, Klaus Oberauer

**Affiliations:** 1 Faculty of Human Sciences, Vinzenz Pallotti University, Vallendar, Germany; 2 Faculty of Psychology, UniDistance Suisse, Brig, Switzerland; 3 Department of Psychology, Cognitive Psychology Unit, University of Zurich, Zurich, Switzerland; 4 University Research Priority Program (URPP), Dynamics of Healthy Aging, University of Zurich, Zurich, Switzerland; 5 Department of Experimental Psychology, University College London, London, UK

**Keywords:** Executive functions, Cognitive control, Individual differences, Hierarchical Bayesian Wiener diffusion model, Structural equation modeling

## Abstract

Attentional control refers to the ability to maintain and implement a goal and goal-relevant information when facing distraction. Previous research has failed to substantiate strong evidence for a psychometric construct of attentional control. This could result from two methodological shortcomings: (a) the neglect of individual differences in speed–accuracy trade-offs when only speed or accuracy is used as dependent variable, and (b) the difficulty of isolating attentional control from measurement error. To overcome both issues, we combined hierarchical Bayesian Wiener diffusion modeling with structural equation modeling. We reanalyzed six datasets that included data from three to eight attentional-control tasks, and data from young and older adults. Overall, the results showed that measures of attentional control failed to correlate with each other and failed to load on a latent variable. Therefore, limiting the impact of differences in speed–accuracy trade-offs and of measurement error does not solve the difficulty of establishing attentional control as a psychometric construct. These findings strengthen the case against a psychometric construct of attentional control.

For more than 20 years, individual-differences researchers have put forward attentional control as a cognitive psychometric construct (e.g., Engle et al., 1999; Miyake et al., 2000). This construct—also referred to as attention control, cognitive control, executive control, executive attention, or executive functions—refers to a person’s ability to maintain and implement a goal and goal-relevant information in the face of distraction (von Bastian et al., 2020). This ability is meant to be general, contributing to success in many different tests and situations demanding attentional control. As such, it should be measurable as a latent variable (i.e., factor) that represents the shared variance of multiple empirical measures of attentional control. The key prerequisite for establishing such a psychometric construct is finding substantial positive correlations between multiple measures of the construct. However, previous research has failed to substantiate strong evidence for the existence of such a psychometric construct (see, e.g., Karr et al., 2018; Rey-Mermet et al., 2019; von Bastian et al., 2020). Proponents of the attentional-control construct have argued that this state of affairs results from two methodological issues (see, e.g., Draheim et al., 2019): (a) the difficulty of isolating attentional control from measurement error, and (b) the neglect of individual differences in speed–accuracy trade-offs. The purpose of the present study was to establish attentional control as a psychometric construct when both issues were overcome. To this end, we combined hierarchical Bayesian Wiener diffusion modeling with structural equation modeling (SEM).

## Why is it difficult to establish attentional control at the latent-variable level?

At first glance, individual differences research was successful in establishing attentional control as a latent variable (Chuderski, 2014; Engle et al., 1999; Friedman et al., 2006, 2008; Friedman & Miyake, 2017; Kane et al., 2004; McCabe et al., 2010; Miyake et al., 2000; Redick et al., 2016; Schweizer & Moosbrugger, 2004; Schweizer et al., 2005; Stahl et al., 2014; Unsworth et al., 2010, 2014, 2015, 2021; Unsworth & McMillan, 2014; Unsworth et al., 2009a, 2009b; Unsworth & Spillers, 2010; see Unsworth et al., 2024, for a recent meta-analysis). However, recent research has put this conclusion into question (see Rey-Mermet et al., 2019; Schubert & Rey-Mermet, 2019; von Bastian et al., 2020, for overviews). First, in some studies, attentional-control tasks did not correlate consistently with each other (e.g., De Simoni & von Bastian, 2018; Guye & von Bastian, 2017; Paap & Greenberg, 2013; von Bastian et al., 2016), and in some cases they failed to load on a factor (Klauer et al., 2010; Krumm et al., 2009) or they had to be merged with other tasks to load on a factor (e.g., Brydges et al., 2012; Hedden & Yoon, 2006; Klauer et al., 2010; van der Sluis et al., 2007). Second, when a factor was established, it was often not coherent (e.g., Chuderski, 2015; Chuderski & Necka, 2012; Chuderski et al., 2012; Hull et al., 2008; Kane et al., 2016; Pettigrew & Martin, 2014; Shipstead et al., 2014; Unsworth et al., 2009a, 2009b). That is, one task’s factor loading was substantially higher than the loadings of the remaining tasks. Thus, the factor represented predominant variance in one task, rather than common variance across multiple measures (see Rey-Mermet et al., 2018, 2019, 2020). Third, a reanalysis of previous datasets suggests a bias toward the publication of well-fitting but nonreplicable structural models of attentional control (Karr et al., 2018).

Proponents of the attentional-control construct have put forward two methodological reasons to explain the difficulty of establishing attentional control at the latent-variable level. The first reason is the contamination of attentional-control measures with measurement error (e.g., Hedge et al., 2018a, 2018b). This problem is severe because these measures must isolate attentional control from other sources of variance in task performance (e.g., intelligence, mental speed). To do so, attentional-control measures typically rely on measuring the performance difference between two conditions, one with high and one with low demand on attentional control. For example, in a color Stroop task, participants have to name the color of color words while ignoring the word meaning. The usual approach for isolating attentional control is by computing the congruency effect—that is, by subtracting the mean RT on baseline trials (e.g., congruent trials, such as the word “red” printed in red for the color Stroop task) from the mean RT on trials with high demand on attentional control (i.e., incongruent trials, such as the word “green” printed in red). The problem with this approach is that there is much trial-by-trial variability in RTs, adding measurement noise to the mean RT that is unknown and unaccounted for (Rouder & Haaf, 2019; Rouder et al., 2023). Taking the difference of two—typically highly correlated—mean RTs removes much of the systematic individual-differences variance while compounding the measurement noise. Therefore, RT differences between congruent and incongruent conditions often have poor reliability (e.g., Hedge et al., 2018a, 2018b).

This shows that there is the need for a better approach of isolating true attentional-control variance. One approach used to bypass computing a difference score in SEM has been to apply a bifactor modeling approach. In this approach, two factors are modeled: (a) a general or common factor on which all trials with both low and high demand on attentional control are forced to load, and (b) a specific attentional-control factor on which only trials with high demand on attentional control are forced to load. The general or common factor is thus meant to extract all individual differences in non-attentional-control processes, whereas the factor extracting individual differences in attentional-control processes is the specific attentional-control factor. The results of several such modelling efforts showed, however, no coherent specific factor of attentional control, thus further emphasizing the difficulty of establishing attentional control at the latent-variable level even when difference scores were not used (e.g., Keye et al., 2009; Rey-Mermet et al., 2019, 2020).

A second reason for the difficulty of establishing attentional control at the latent-variable level is that most attentional-control measures rely exclusively on response times, so that individual differences in speed–accuracy trade-offs are disregarded (Draheim et al., 2019). Some participants might favor speed over accuracy, while others favor accuracy over speed. Hence, a measure that ignores individual differences in accuracy could miss a substantial part of the variance in attentional-control ability. One way to consider individual differences in speed–accuracy trade-offs is to integrate both dependent measures (i.e., RTs and accuracy) into a single score. Unfortunately, although these scores are relatively easy to compute, they are generally not able to unambiguously account for differences in speed–accuracy trade-offs (e.g., Liesefeld & Janczyk, 2019; Vandierendonck, 2017). It has even been put forward that these scores reflect speed–accuracy trade-offs, making them difficult to interpret (Hedge et al., 2018a, 2018b).

Another way to take into account speed–accuracy trade-offs is to push all attentional-control variance into accuracy—for example, by measuring attentional control under a time limit, which is calibrated according to each person’s performance. With such a deadline approach, low attentional-control ability would be measured as low accuracy, irrespective of whether speed is favored over accuracy or accuracy is favored over speed. Previous research has implemented this response deadline approach in different ways (Draheim et al., 2021, 2024; Rey-Mermet et al., 2019). For example, Draheim et al. (2021) used a response deadline, which changed after every 16 trials. That is, if the responses were correct on at least 15 trials, the response deadline decreased, thus providing less time to respond. In contrast, if fewer than 15 trials were correct, the response deadline increased, thus providing more time to respond. The change occurred based on performance of both incongruent and congruent trials. In that study, attentional control was measured as the response deadline after the final block. In a follow-up study, Draheim et al. (2024) adjusted their response deadline approach in two ways. First, the response deadline changed after every incongruent trial, depending on the performance on this trial only. Second, attentional control was measured as the average response time of the final four trials in which the response deadline reversed (i.e., increased after having decreased or decreased after having increased). In both studies, the results suggest well-fitting models including coherent factors. However, in these studies, the dependent variable measures how quickly and accurately a person can do the task regardless of congruency condition. Therefore, it is not apparent why the measures should capture attentional control and how these measures remove variance of other processes, which are not attentional-control processes.

Rey-Mermet et al. (2019) overcame these issues by applying the response deadline on trials with only one relevant response feature (so-called neutral trials, such as, e.g., a row of red *X*s for the color Stroop task). These trials were performed in the first block of the attentional-control task. In the subsequent blocks, incongruent and congruent trials were presented, and the response deadline was fixed to the calibrated duration. Attentional control was thus measured as the difference in error rates between incongruent and congruent trials. With such a response deadline approach, Rey-Mermet et al. (2019) reduced the individual differences in general ability and in the ability to carry out all the non-attentional-control processes. SEM, however, identified no models with good fit statistics and coherent factors. Therefore, when differences in speed–accuracy trade-offs are taken into account with a response deadline approach and when non-attentional-control processes are controlled for, it is still difficult to establish attentional control at the latent-variable level.

Nevertheless, the response deadline approach may affect how participants engage with the tasks. As a consequence, for all studies in which the tasks were implemented with a response deadline approach (Draheim et al., 2021, 2024; Rey-Mermet et al., 2019), this could raise doubts about the validity of these tasks as measures of attentional control.

## How to overcome the contamination of attentional-control measures with measurement error and the neglect of individual differences in speed–accuracy trade-offs?

One approach to address both issues—that is, the contamination of attentional-control measures with measurement error and the neglect of individual differences in speed–accuracy trade-offs—is to employ a cognitive model, the diffusion model (e.g., Donkin & Brown, 2018; Ratcliff & McKoon, 2008), as measurement model. This means that the parameters obtained from the diffusion model are used as measures of the latent variables of interest, such as the ability to control attention.

The diffusion model is a well-validated model of simple decisions that uses the full set of behavioral data to estimate three parameters of theoretical interest: (1) the drift rate, reflecting the rate at which information in favor of one or the other decision accumulates over time, (2) the caution parameter controlling the speed–accuracy trade-off, and (3) the non-decision time capturing primarily the speed of sensory and motor processes. This model addresses the problem of speed–accuracy trade-off in two ways (see Hedge et al., 2018a, 2018b). First, it provides a framework in which error rates as well as the RTs of both correct and incorrect responses are accounted for. Second, it accounts for the effect of speed–accuracy trade-offs by separating the quality of information processing (i.e., the drift rate) from the person’s speed–accuracy setting (i.e., the caution parameter). The diffusion model also addresses the problem of measurement error in two ways. First, the diffusion model is fitted to the joint distribution of RT and accuracy, which thus considerably reduces the effect of individual RTs, and the associated noise, on the parameter estimates compared with mean RTs. Second, the diffusion-model parameters jointly determine the width of the RT distribution, thereby accounting for the trial-by-trial variability of each participant.

So far, only four studies have investigated attentional control by combining a diffusion modeling approach with SEM or at least a correlative approach. In three studies (Löffler et al., 2024; Weigard et al., 2021; Yangüez et al., 2024), the standard diffusion model (Ratcliff, 1978) or a simplified version of this model (Wagenmakers et al., 2007) was applied. In these studies, the drift rates were considered as measures of the efficiency of processing decision-relevant information. Attentional control was thus assessed in three different ways. First, attentional control was measured by subtracting drift rates between low and high attentional-control conditions (Löffler et al., 2024; Weigard et al., 2021; Yangüez et al., 2024). Second, attentional control was measured using only the drift rates of the high attentional-control conditions (Löffler et al., 2024; Yangüez et al., 2024). Third, attentional control was estimated as the remaining variance specific to attentional control after controlling for baseline processes (e.g., processing speed; Löffler et al., 2024; Yangüez et al., 2024). SEM identified no model including a coherent factor of attentional control different from processing speed.^1^

In the last study (Hedge et al., 2022), the diffusion model for conflict tasks (Ulrich et al., 2015)—an extension of the standard diffusion model—was applied to different datasets including two to three Stroop-like tasks. In this model, in addition to the three typical parameters, a rescaled gamma function was included to capture conflict processing (in particular, the assumption that the stimulus features to be ignored have an early impact on the decision processes). This changes the interpretation of the drift-rate parameter which, in this case, reflects the processing efficiency once the conflict has been resolved (rather than the speed of information uptake in the complete decision process, as typically interpreted in the standard diffusion model). Therefore, in this diffusion model, attentional control is no longer captured by the drift rate but by the parameters underlying the rescaled gamma function. Hedge et al. (2022) reported significant zero-order correlations for the parameters reflecting non-conflict processing in their diffusion modeling approach (e.g., meta-analyzed correlation value = 0.32 for the drift rate and meta-analyzed correlation value = 0.54 for the boundary separation). However, the correlations between parameters capturing attentional control (i.e., those underlying the gamma function) were weak (meta-analyzed correlation values = 0.04).

Together, these studies represent a first step by demonstrating the feasibility of combining diffusion models with a correlative approach or SEM. However, these studies have some limitations. First, the study of Hedge et al. (2022) has two shortcomings. One is that the extension of the diffusion model by a conflict-resolution parameter increases the model complexity, and with it the risk of overfitting noise in the data (e.g., van Ravenzwaaij & Oberauer, 2009). The other is that the analyzed datasets included only two to three Stroop-like tasks, and no SEM was computed. This limits the generality of these findings. Second, the studies of Löffler et al. (2024), Weigard et al. (2021), and Yangüez et al. (2024) have their limitation as well: Computing the drift rates separately for trials with low and high demand on attentional control and then subtracting them compounds the measurement error of the two drift-rate estimates in the same way as subtracting mean RTs does. This counteracts the reduction of measurement error that can be achieved by applying the diffusion model. Finally, all the studies have the limitation of applying a two-step procedure (Hedge et al., 2022; Löffler et al., 2024; Weigard et al., 2021; Yangüez et al., 2024). That is, the diffusion model parameters were first estimated, and the correlations were subsequently calculated on these estimates. The two-step procedure ignores the measurement error in the parameter estimates, and thereby underestimates the uncertainty in the estimated correlations between them. Together, this highlights that these studies are not sufficient to conclude whether the impact of speed–accuracy trade-offs and/or the contamination of attentional-control measures by measurement error explain the difficulty of establishing attentional control at the latent-variable level.

## The present study

The purpose of the present study was to determine whether a coherent factor of attentional control can be established when individual differences in speed–accuracy trade-offs and measurement error were accounted for. To this end, we estimated a Wiener diffusion model, as well as the correlations among model parameters, in a hierarchical Bayesian framework (e.g., Donkin & Brown, 2018; Ratcliff & McKoon, 2008; Vandekerckhove et al., 2011). Then, based on these correlations, we computed SEM to determine whether a coherent factor of attentional control can be established. To perform these analyses, we reused six datasets from three studies (Kane et al., 2016; Rey-Mermet et al., 2018; Whitehead et al., 2019).

In the present study, we opted for a hierarchical Bayesian Wiener diffusion model because this modeling approach has three advantages. The first advantage is that it combines the strength of the diffusion model with a hierarchical Bayesian statistical framework to reduce measurement error. Specifically, it takes into account measurement error in three different ways. First, the Wiener diffusion model addresses the problem of measurement error—that is, the noise arising from trial-by-trial variability of RTs—because it explicitly models the shape of the trial-by-trial RT distribution. Second, the hierarchical Bayesian framework (Vandekerckhove et al., 2011) substantially reduces the measurement error in the individual-level effects because the parameter estimates of each individual participant are informed by the data of all other participants (e.g., Rouder & Haaf, 2019). Third, we modeled the variance–covariance matrix of the individual-level effects—the basis for the SEM analysis—as a parameter of the hierarchical model. This joint estimation approach decreased the measurement error in the variance–covariance matrix compared with a two-step procedure in which first the diffusion model parameters are estimated and the correlations are then subsequently calculated from these estimates (cf. Hedge et al., 2022; Löffler et al., 2024; Weigard et al., 2021; Yangüez et al., 2024). Furthermore, because we implemented our model in Stan (Carpenter et al., 2017), we could use a non-informative LKJ prior for the correlation matrix as well as independent weakly informative priors for the variances. The non-informative LKJ prior has two benefits over common alternatives in Bayesian approaches, such as a (scaled) inverse-Wishart prior for the variance–covariance matrix (e.g., JAGS; Plummer, 2003; Rouder & Haaf, 2019; see Gelman & Hill, 2007): It is conceptually and computationally simpler and it is less likely to have an effect on the estimation of the variance–covariance matrix (see, e.g., Klauer, 2010).

The second advantage of our hierarchical Bayesian Wiener diffusion model is that it can be applied to the broad variety of tasks used in all the datasets we selected (Rey-Mermet et al., 2018; Whitehead et al., 2019). This is, for example, not the case for the diffusion model for conflict tasks. This diffusion model was specifically developed to account for the flanker and Simon tasks (Ulrich et al., 2015) and then recently extended to Stroop tasks (Ambrosi et al., 2019; Hedge et al., 2019, 2022), but it cannot be applied to all tasks we consider.

The third advantage of using the Wiener diffusion model, and not a more complicated diffusion model, such as the diffusion model for conflict tasks, is that choosing a simpler model variant strikes a good compromise in the statistical *bias-variance trade-off* (e.g., Yarkoni & Westfall, 2017). Any statistical model needs to strike a balance between the ability to adequately describe the signal in the data and at the same time avoid overfitting the noise. Our model accounts for the main pattern in the data that are relevant for our research question (i.e., the impact of the speed–accuracy trade-off and measurement error). A more complex diffusion model may be able to account for some more subtle data patterns of some of the tasks (i.e., achieve lower bias), but is more likely to overfit (i.e., higher variance; White et al., 2018). This is in line with theoretical and empirical results showing that simpler diffusion models outperform complex diffusion models in terms of predictive ability and parameter stability (Boehm et al., 2018; Dutilh et al., 2019; van Ravenzwaaij & Oberauer, 2009).

To sum up, the present study goes beyond previous research by investigating attentional control with a diffusion modeling approach (Hedge et al., 2022; Weigard et al., 2021) in two ways. First, in contrast to Hedge et al. (2022) who applied the diffusion model for conflict tasks, we applied a Wiener diffusion model to overcome the risk of overfitting noise in the data. Applying the Wiener diffusion model has also the advantage that we can use our approach on a larger number of attentional-control tasks. Second, in contrast to previous research (Hedge et al., 2022; Löffler et al., 2024; Weigard et al., 2021; Yangüez et al., 2024) in which the diffusion model parameters were first estimated and then the correlations were computed, we applied a joint estimation approach in which the correlations are modeled as a parameter of the hierarchical model. This approach has the advantage that it decreased the measurement error in comparison to the two-step procedure used in previous research to estimate the correlations.

### How did we combine the hierarchical Bayesian diffusion model with SEM?

Estimating the hierarchical Bayesian diffusion model produced a posterior distribution of the variance–covariance matrix of the diffusion-model parameters across tasks. Then, we applied SEM to the posterior distribution of the variance–covariance matrix. We did this once with the posterior means (as point estimates of the variances and covariances), and additionally multiple times with samples from the posterior distribution to gauge what the uncertainty in the posteriors implies for the uncertainty of SEM fits and SEM parameter estimates. Moreover, we used two approaches to isolate the attentional-control variance. Following the predominant practice in previous research, we used a *difference coding* approach to estimate parameter differences between the two conditions (i.e., the difference between trials with low and high demand on attentional control). To avoid the measurement error from the subtraction of drift rates, we directly modeled a difference parameter representing the mean difference between trials with low and high demand on attentional control. In addition, to overcome the problems associated with difference scores, we employed a *condition coding* approach, which starts from diffusion-model parameters in each condition and uses a bifactor SEM to extract attentional-control variance.

### What are we expecting?

We hypothesized that if the difficulty of establishing attentional control as a psychometric construct results from the neglect of individual differences in speed–accuracy trade-offs and/or the large measurement error in attentional-control measures, we would find a coherent factor of attentional control. The reason is that we employed a state-of-the-art computational approach for addressing these two issues: The Wiener diffusion model accounts for speed–accuracy trade-offs in a theoretically principled way, and our hierarchical Bayesian implementation estimated the variance–covariance matrix—the basis for the SEM—as a set of latent model parameters separated from trial-by-trial noise, thereby minimizing the influence of measurement error. In contrast, if the difficulty of establishing attentional control as a psychometric construct reflects the true nature of attentional control—namely, that a *general* ability to control attention does not exist—then we would expect to find no coherent factor of attentional control.

We expected to establish a coherent factor of attentional control on the drift-rate parameter. The reason is that, in the diffusion model we used, the drift rate represents the rate at which information for the correct response is accumulated in a given trial (e.g., Ratcliff & McKoon, 2008). Thus, the drift rate captures the efficiency of processing decision-relevant information. In attentional-control tasks, the ability to control attention is assumed to play a primary role in how well individuals can extract information from the relevant aspect of the stimulus to select the correct response in the face of potent distractors, such as in an incongruent trial (e.g., Hübner et al., 2010; Ridderinkhof, 2002; Verguts & Notebaert, 2009; Wiecki et al., 2013). Therefore, a higher ability to control attention should contribute to a higher drift rate in high-conflict conditions. This implies that relative to a condition with low demand on attentional control, the drift rate should be reduced in the condition with high demand on attentional control because the irrelevant and misleading information of this condition pushes the accumulator toward the wrong response. Moreover, an individual with better attentional-control ability should show a smaller reduction of drift rates between both conditions because of their better ability to prevent irrelevant and misleading information from affecting the drift rate.

If a coherent factor is established not on the drift rate but rather on the caution or non-decision-time parameters, this would not support attentional control as a psychometric construct. The reason is that these parameters reflect aspects of performance that are theoretically unrelated to attention control ability—namely, as a person’s speed–accuracy trade-off preference—and the speed of sensory and motor processes, respectively.

## Method

### Data set selection

We did not aim for a comprehensive reanalysis of all published studies with which we could investigate our research question. Rather, we chose a number of suitable datasets based on the following four inclusion criteria. First, each dataset included a minimum number of three conflict tasks, such as the Stroop task. We opted for these conflict tasks for four reasons. First, in most previous individual-differences research, conflict tasks were used to assess attentional control (see Rey-Mermet et al., 2019; von Bastian et al., 2020, for overviews of the tasks used in previous research). Second, conflict tasks have been assumed to measure the core ability necessary for attentional control (Miyake & Friedman, 2012). Third, the conflict tasks are the tasks for which critics have emphasized the neglect of individual differences in speed–accuracy trade-offs and the difficulty of isolating attentional control from measurement error (Draheim et al., 2019, 2021, 2024). Finally, nearly all previous research with the diffusion-modeling approach has applied it to conflict tasks (Hedge et al., 2022; Löffler et al., 2024; Weigard et al., 2021; Yangüez et al., 2024). We set three tasks as the minimum because that number is necessary to estimate a latent factor in structural equation modeling. Furthermore, each task should include incongruent and congruent trials. The reason is that both types of trials are required in order to model attentional control as the variance that is left in incongruent trials once baseline performance in congruent trials has been accounted for. Without taking this into consideration, the measure would lack construct validity, thus making it unclear whether the measure assesses attentional control or other processes (Löffler et al., 2024).

The second inclusion criterion requires that attentional control was measured with both reaction times and error rates. This criterion is necessary to allow the application of the diffusion model. This leads, however, to the exclusion of studies using a response deadline approach (Draheim et al., 2021, 2024; Rey-Mermet et al., 2019). In those studies, the reaction-time distribution is censored, which can result in biased estimates for the diffusion model.

The third inclusion criterion concerns the availability of trial-level raw data with an appropriate documentation. This criterion is necessary because our approach relies on data at the trial level.

The last inclusion criterion concerns the diversity of the datasets regarding the sample sizes, the numbers of trials and the labs where the data were collected. Thus, we selected datasets with different sample sizes, different number of trials and coming from different labs in order to assess the generality and robustness of the results obtained with our approach.

### Tasks

We reanalyzed eight tasks (i.e., the color Stroop, number Stroop, arrow flanker, letter flanker, Simon, global–local, positive compatibility, and negative compatibility tasks) from Rey-Mermet et al. (2018), three tasks (i.e., the color Stroop, spatial Stroop,^2^ and flanker tasks) from Whitehead et al. (2019), and the number Stroop, spatial Stroop, arrow flanker, and letter flanker tasks from Kane et al. (2016). These were the tasks for which the attentional-control measure was the difference between congruent and incongruent trials, so the same diffusion model could be applied to all tasks. The tasks are shortly described in Table 1. A complete description of the tasks (including material and procedure) can be found in Rey-Mermet et al. (2018), in Whitehead et al. (2019), and in Kane et al. (2016), respectively.

**Table 1:** Tasks analyzed in the present study

Task	Decision	Sample size
Tasks from Rey-Mermet et al. (2018)^a^		
Color Stroop	Color words (i.e., the words “red” “blue,” “green,” or “yellow”) or row of four *X*s were printed either in red, blue, green, or yellow. Color words were either incongruent to the meaning of the words (e.g., the word “green” printed in green), congruent (e.g., the word “red” printed in red), or neutral (e.g., XXXX printed in green). Participants were instructed to indicate the color of color words while ignoring the meaning of the words	Dataset 1 (Young adults): 116 Dataset 2 (Older adults): 125
Number Stroop	One to four digits or symbols were displayed centrally. The number of digits could be incongruent to the numerical value (e.g., 111), congruent (e.g., 333), or neutral (e.g., $$$). Participants were instructed to count the number of characters while ignoring the numerical value of digit characters	Dataset 1 (Young adults): 120 Dataset 2 (Older adults): 128
Arrow flanker	Five arrows were presented centrally. The direction of the central arrow was either incongruent to the four flanking arrows (e.g., ← ← → ← ←), congruent (e.g., → → → → →), or neutral (e.g., –– → ––). Participants were asked to respond to the direction of the central arrow (left or right) while ignoring the four flanking characters	Dataset 1 (Young adults): 117 Dataset 2 (Older adults): 138
Letter flanker	Five letters were presented centrally. Participants were asked to decide whether the central letter was a vowel (*E* or *U*) or consonant (*S* or *H*) while ignoring the four flanking characters. The response to the central letter was either incongruent to the response of the flanking letters (e.g., SSESS), congruent (UUEUU or EEEEE), or neutral (e.g., ##E##)	Dataset 1 (Young adults): 118 Dataset 2 (Older adults): 140
Simon	A square or a circle was presented either on the left or right side of the screen or centrally. Participants were asked to indicate the shape (square vs. circle) by using manual responses (a left or a right response key) while ignoring the location of the shape on the screen. The location of the shape on the screen was either incongruent to the location of the response key (e.g., a square presented on the right side but requiring pressing the left key), congruent (e.g., a square presented on the left side and requiring pressing the left key), or neutral (e.g., a square presented centrally but requiring pressing the left key)	Dataset 1 (Young adults): 119 Dataset 2 (Older adults): 140
Global-local^b^	Element letters forming a large letter (e.g., a large *Y* built from small *V*s) were presented. In the global version of the task, participants were asked to identify the global letter (i.e., the large *Y*) while suppressing the response induced by the local elements. In the local version of the task, participants were asked to identify the local elements (i.e., the small *V*s) while suppressing the response induced by the global letter. The local elements were either incongruent to the global letter (e.g., a large *Y* built from small *V*s), congruent (e.g., a large *V* built from small *V*s), or neutral (e.g., a large *Z* built from small *V*s)	Dataset 1 (Young adults): 118 Dataset 2 (Older adults): 131
Positive compatibility	A prime (e.g., “<< ,” “ >>,” or “ == ”) was first presented. To prevent conscious identification, the prime was followed by a mask (which consisted of the overlap of all prime exemplars). A target arrow (i.e., “<< ” or “ >>”) was then displayed either above or below the mask. Participants were asked to indicate the direction of the target arrow (i.e., left or right) while ignoring the information induced by the prime. The prime and the arrow were either incongruent (e.g., the prime “<<” followed by the target “>>”), congruent (e.g., the prime “<<” followed by the target “<<”), or neutral (e.g., the prime “==” followed by the target “>>”)	Dataset 1 (Young adults): 114 Dataset 2 (Older adults): 130
Negative compatibility	This task was similar to the positive compatibility task, except that a delay of 150 ms was introduced between the prime and target. During the delay, the prime is assumed to induce a response, which is then automatically suppressed. However, this response needs to be reactivated when the target and the prime require the same response (i.e., in congruent trials). The negative compatibility effect reflects the time cost of reactivating the primed response after it has been suppressed. Congruent trials are thus the trials assumed to induce attentional control, while incongruent trials are baseline trials	Dataset 1 (Young adults): 118 Dataset 2 (Older adults): 132
Tasks from Whitehead et al. (2019)^c^		
Color Stroop	This task was similar to the color Stroop task used by Rey-Mermet et al. (2018)	Dataset 3 (Experiment 1): 178 Dataset 4 (Experiment 2): 195 Dataset 5 (Experiment 3): 210
Spatial Stroop	Directional words (i.e., the words “right,” “left,” “up,” or “down”) were printed either in the right, left, top, and bottom part of the screen. Directional words were either incongruent to the location of the words (e.g., the word “right” presented in the top part) or congruent (e.g., the word “up” presented in the top part). Participants were instructed to respond to the direction word while ignoring its location	Dataset 3 (Experiment 1): 178 Dataset 4 (Experiment 2): 195 Dataset 5 (Experiment 3): 210
Flanker	Five letters (i.e., *D*,* F*,* J*, and *K*) were presented centrally. Participants were asked to identify the central letter while ignoring the four flanking letters. The response to the central letter was either incongruent to the response of the flanking letters (e.g., JJDJJ) or congruent (DDDDD)	Dataset 3 (Experiment 1): 178 Dataset 4 (Experiment 2): 195 Dataset 5 (Experiment 3): 210
Tasks from Kane et al. (2016)^d^		
Number Stroop	This task was similar to the color Stroop task used by Rey-Mermet et al. (2018), except for the following two modifications. First, there were no neutral trials. Second, 80% of the trials were congruent whereas 20% were incongruent	Dataset 6: 437
Spatial Stroop	Directional words (i.e., the words “right,” “left,” “above,” or “below”) and asterisks were printed either in the right, left, top, and bottom part of the screen. In addition, words were presented either left, right, above, or below the asterisks. Participants were instructed to respond to the relative position of the word to the asterisk while ignoring the identity of the word and its absolute location. Directional words were either incongruent to both the absolute and relative location of the words (e.g., the word “left” presented to the right of the asterisk and both presented to the right of the fixation) or congruent to both the absolute and relative location of the words and (e.g., the word “left” presented to the left of the asterisk and both presented to the left of the fixation). There were also trials in which the words were congruent for the absolute location but incongruent for the relative position (e.g., the word “left” presented to the right of the asterisk and both presented to the left of fixation)	Dataset 6: 412
Arrow flanker	This task was similar to the arrow flanker task used by Rey-Mermet et al. (2018), except for the following modifications. First, the neutral trials consisted of a target arrow amid dots. Second, there were trials in which the target was presented in the middle of upward-pointing arrows	Dataset 6: 396
Letter flanker	Seven letters or characters were presented centrally. Participants were asked to respond to the direction of the middle *F* (normal vs. backward). The response to the central letter was either incongruent to the response of the flanking letters or congruent (e.g., FFFFFFF). In addition, there were trials in which the *F* was presented in the middle of dots (neutral trials) and trials in which the target was presented in the middle or right- and left-facing *E*s and tilted *T*s	Dataset 6: 395

In all tasks used by Rey-Mermet et al. (2018), there were two types of baseline trials. In nearly all tasks (see the negative-compatibility task for an exception), the baseline trials were (1) the congruent trials in which there was no conflict between stimulus or response features, and (2) the neutral trials in which there was only one response-relevant feature. Only in the negative compatibility task, the two types of baseline trials are incongruent and neutral trials. In all tasks used by Whitehead et al. (2019), there was only one type of baseline trials, that is, the congruent trials. In all tasks used by Kane et al. (2016), congruent and incongruent trials were presented. We used the congruent trials as baseline trials. Depending on the task, there were, however, additional types of trials. Critically, all trials were used to estimate the correlation matrices in the hierarchical Bayesian Wiener diffusion modeling approach for the condition coding

^a^Rey-Mermet et al. (2018) tested young and older adults, whose data are referred to as Dataset 1 and Dataset 2 in the present study. In addition to the tasks presented in the table, all participants performed an antisaccade task, a stop-signal task, and a task assessing n-2 repetition cost. These tasks were not analyzed in the present study because no diffusion modeling can be applied to them

^b^Similar to Rey-Mermet et al. (2018), we only analyzed the local version of this task because substantial difference scores are predominantly observed in this version of the task

^c^The study from Whitehead et al. (2019) consists of three experiments, whose data are referred to as Dataset 3, Dataset 4, and Dataset 5 in the present study. In Dataset 3, the experiment was so designed that congruent and incongruent trials were presented equally often. These trials were presented in an alternating-presentation design splitting a four-choice, four-response task into two groups (i.e., two choice, two response). Thus, the trials of both groups are presented in alternating order, preventing any feature repetition. In Dataset 4, the design of the experiment was similar to the first experiment, except that congruent trials were presented in 25% of the trials, whereas incongruent trials were presented in 75% of the trials. In Dataset 5, the experiment was so designed that congruent and incongruent trials were presented equally often, and the trials were presented in a typical four-alternative forced choice button-press task (i.e., without an alternating-presentation design)

^d^In addition to the tasks presented in the table, participants in Kane et al. (2016) performed tasks used to measure working memory and thought probes. Critically, they also performed further attentional-control tasks (i.e., two antisaccade tasks, a go/no-go task, a cued search task, and a masked flanker task). The antisaccade, go/no-go, and cued search tasks could not be analyzed because they did not include a congruent and an incongruent condition, or comparable conditions with low vs. high conflict. In the masked flanker task, there was a very short response-deadline (1 s), which both censored the data to an unacceptably large degree and created a very high error rate. Thus, no diffusion modeling could be applied

### Datasets and data preparation

#### Two datasets from Rey-Mermet et al. (2018)

For the reanalysis of Rey-Mermet et al.’s (2018) data, we considered the data from young and older adults as two distinct datasets (i.e., Dataset 1 and Dataset 2, respectively). Specifically, we used the data from 120 young adults and 143 older adults—that is, the full sample from the original study after removing participants that did not meet their psychiatric or demographic inclusion criteria or for which some error occurred during testing.

To prepare the data for a diffusion model analysis, we removed, on a by-task basis, the data from participants for which more than 2.5% of the trials were outside the response window of 2 s. This procedure removed a total of 4.6% of trials. The reason for this is that responses outside this window were not recorded (i.e., the RT distribution was censored) and applying the diffusion model to an RT distribution where a non-negligible part of the distribution is censored can lead to biased estimates. The final sample size for each task is presented in Table 1. On a by-trial basis, we further excluded 0.15% of trials where either the computer malfunctioned, participants used an illegitimate response key, or responded too fast (i.e., faster than 200 ms). We also removed the remaining 0.1% of trials with responses outside the 2-s response window.

#### Three datasets from Whitehead et al. (2019)

For the reanalysis of Whitehead et al.’s (2019) data, we used each of their three experiments as a separate dataset (i.e., Dataset 3 designates the data from Experiment 1, Dataset 4 designates the data from Experiment 2, and Dataset 5 designates the data from Experiment 3). We used the same participants as analyzed in the original study (i.e., 178 participants in Dataset 3, 195 participants in Dataset 4, and 210 participants in Dataset 5). Because Whitehead et al. (2019) did not employ a response window, we did not need to specifically prepare the data for diffusion modelling and used the same trial-based exclusion criteria as in the original study (i.e., excluded responses smaller than 0.2 s and larger than 3 s). This results in the exclusion of 1.0% of trials in Dataset 3, 1.6% of trials in Dataset 4, and 1.4% of trials in Dataset 5.

#### One dataset from Kane et al. (2016)

For the reanalysis of Kane et al.’s (2016) data, we initially included the same 472 participants as analyzed in the original study. We prepared the data for a diffusion model analysis similar to the previous datasets. First, we applied a response window so that we excluded responses smaller than 200 ms but larger than 1.5 s for the arrow flanker task and 3 s for the other tasks. The upper limit of 1.5 s for the arrow flanker task and the upper limit of 3 s for the letter flanker task were given because responses outside this window were not recorded (see Kane et al., 2016). For the two other tasks (i.e., Stroop tasks), we used the same upper limit as the one we used for the datasets from Whitehead et al. (2019). Removing responses smaller than 200 ms resulted in the exclusion of 0.5% trials or less for each task. Removing responses larger than the upper limit (i.e., 1.5 s for the arrow flanker task and 3 s for the other tasks) resulted in the exclusion of 0.8% trials in the arrow flanker task and less than 0.5% in all other tasks. Similar to the data of Rey-Mermet et al. (2018), we then removed, on a by-task basis, the data from participants for which more than 2.5% of the trials were outside the response window. This resulted in the exclusion of 7.4% of trials. Finally, because some participants had accuracy that was both below chance and much lower than the average accuracy in some conditions, we excluded a further 20 participant-by-task combinations. This was done because we did not want individual participants to have an outsized influence on the results. After exclusion, we were left with data from 443 participants that provided responses in at least two tasks. The number of participants per task is given in Table 1.

### Data analysis

Data were analyzed using R (R Core Team, 2022). We used the following packages: *brms* (Bürkner, 2017), *lavaan* (Rosseel, 2012), *psych* (Revelle, 2021), *semTools* (Jorgensen et al., 2021), *DescTools* (Signorell et al., 2020), and *ggplot2* (Wickham et al., 2021).

#### Diffusion modeling

We used a hierarchical Bayesian variant of the Wiener diffusion model (Vandekerckhove et al., 2011) with an accuracy coding. This model accounts for the entire data (i.e., RT distributions of correct and error trials) with three latent parameters: (a) the drift rate, a measure of the efficiency of information processing in the decision process, (b) the boundary separation, a measure of response caution that controls the speed–accuracy trade-off, and (c) the non-decision time. The model was applied separately to all individual choices and associated RTs across tasks for each dataset (i.e., Datasets 1 to 6). Furthermore, we applied two different parametrizations—a difference-coding parametrization and a condition-coding parametrization—to each dataset.

In the *difference-coding* parametrization, we only considered the congruent and incongruent trials. For the drift rate, we estimated two fixed-effect parameters per task: (a) the mean in the congruent trials and (b) a difference parameter representing the mean difference between congruent and incongruent trials. This difference parameter was our main measure of attentional control. In the *condition-coding* parametrization, we considered all trial types (i.e., incongruent, congruent, and neutral trials in Datasets 1 and 2, incongruent and congruent trials in Datasets 3 to 5, and incongruent, congruent, neutral and other trials in Dataset 6). For the drift rate, we estimated a fixed-effect parameter per trial type. For example, a mean drift rate was computed separately for incongruent, congruent, and neutral trials in Datasets 1 and 2, and for incongruent and congruent trials in Datasets 3 to 5. For both parametrizations, we estimated one fixed-effect parameter per task for the boundary separation and for the non-decision time, respectively.

In addition to the fixed-effect (or group-level) parameters, we estimated by-participant random-effects (i.e., the variance of parameters over participants), the correlations among all random-effects for each fixed-effect parameter, and individual-level (displacement) parameters. This resulted in quite large models. An overview of the numbers of observations and estimated parameters is presented in Table 2. The numbers of observations used for estimation across datasets and parameterizations varied between approximately 150,000 and 445′000. The total number of estimated parameters varied between approximately 5,000 and 14,500 and the number of estimated correlation parameters (which were of central importance) varied between 21 and 990.

**Table 2:** Model overview

Dataset	*N*	Total observations	*O*_*t*_ (mean)	*O*_*t*_ (min)	*O*_*t*_ (max)	*P* (diff)	*C* (diff)	*V* (diff)	*P* (cond)	*C* (cond)	*V* (cond)
1	120	157,737 / 236,571	149 / 223	55 / 82	192 / 288	5,023	630	36	6,481	990	45
2	143	177,903 / 266,877	150 / 225	48 / 72	192 / 288	5,815	630	36	7,471	990	45
3	178	402,414	754	634	762	6,526	21	12	6,708	66	12
4	195	445,293	761	63	1534	7,138	21	12	7,320	66	12
5	210	401,669	638	601	1732	7,678	21	12	7,860	66	12
6	443	161,290 / 233,309	98 / 142	61 / 117	149 / 172	7,162	40	16	14,530	288	32

*N* = Number of participants; Total observations = Overall number of trials; *O*_*t*_ = Number of observations/trials per task. For Datasets 1, 2, and 6, values before the “/” indicate number for the difference coding parameterization and values after the “/” for the condition coding parameterization (for Datasets 3 to 5, the values were the same, as they did not include neutral trials). *P* = Total number of estimated parameters; *C* = Number of estimated correlation parameters; *V* = Number of estimated fixed effects parameters and number of estimated variance parameters. The number of individual-level displacement parameters (i.e., the random-effects) is given by *P* – *C* – 2* V*. diff = difference coding parameterization; cond = condition coding parameterization. For the difference coding parameterization of Datasets 3 to 5, we only estimated correlation parameters among each diffusion parameters but not across diffusion parameters (e.g., only among all drift rates, but not between drift rates and boundary separation) to decrease estimation time

We estimated the models using *brms* (Bürkner, 2017), which uses the probabilistic programming language Stan (Carpenter et al., 2017) for model estimation. This allowed us to estimate the variance–covariance matrix in two parts—a correlation matrix and a variance vector. For the correlation matrix, we used a non-informative LKJ prior with concentration parameter = 1, and for the variances a weakly informative *t*-distribution prior ($df=3, \text{scale}=10$). All other priors were also weakly informative (i.e., Cauchy with scale of 5 for mean drift-rate parameters for both coding parametrizations, and with scale of 2.5 for drift-rate difference parameters for difference coding). We estimated all diffusion-model parameters and correlations on the unconstrained real line (i.e., no link functions). In other words, we assumed multivariate normal distributions for the individual-level parameters. The brms syntax for estimating each model can be found in the supplemental materials on OSF.

We initially estimated each model using six (Datasets 1 to 5) or four (Dataset 6) independent chains with 500 warmup samples per chain and retained a further 500 post-warmup samples. If chain convergence was not good (i.e., $\widehat{R}<1.05$) for all random-effect parameters, variance parameters, and correlations relevant for our research question we refit the model with more post-warmup samples until convergence was reached. The model requiring the largest number of samples to reach convergence was the condition coding model for Dataset 6 with 4,500 post-warmup samples. Estimating each model took several weeks on a high-performance desktop computer (in case a model required multiple refits, this resulted in fitting times of a few months for individual datasets).

#### Structural equation modeling

For each dataset, we first estimated a series of SEMs using the posterior means of each value in the variance–covariance matrix of the diffusion-model parameters (each analysis only used a subset of the full covariance matrix; e.g., only the covariances among the difference parameters of the drift rate). Then, we fitted the same series of SEMs separately to 500 variance–covariance matrices sampled from the posterior distribution (i.e., these 500 samples were a subset of all post-warmup draws). The latter analysis takes into account the uncertainty in the estimate of the covariance matrix, which depends in part on the measurement error in the data.

Model fit was evaluated via multiple fit indices (Hu & Bentler, 1998, 1999): The χ^2^ goodness-of-fit statistic, Bentler’s comparative fit index (CFI), the standardized root mean square residual (SRMR), and the root mean square error of approximation (RMSEA). For the χ^2^ statistic, a small, non-significant value indicates good fit. For the CFI, values larger than 0.95 indicate good fit, and values between 0.90 and 0.95 indicate acceptable fit. SRMR values smaller than 0.08 indicate good fit. RMSEA values smaller than 0.06 indicate good fit, and values between 0.06 and 0.08 indicate acceptable fit. However, as the RMSEA tends to over-reject true population models at smaller sample size (i.e., smaller than 250; Hu & Bentler, 1998), this fit index was not taken into account when the sample size was smaller than 250 participants. In this case, it was only provided for the sake of completeness.

In addition, the following criteria had to be met for a model to be considered an adequate representation of a latent variable: (1) the Kaiser–Meyer–Olkin (KMO) index—a measure of whether the correlation matrix is factorable—should be larger than 0.60 (Tabachnick & Fidell, 2019); (2) most of the error variances should be lower than 0.90; (3) most of the factor loadings should be larger than 0.30; (4) no factor should be dominated by a large loading from one task; (5) the amount of shared variance across tasks—that is, “factor reliability” as assessed by the coefficient ω for the single-factor models (Raykov, 2001), and the hierarchical omega ω_h_ for the bifactor models (McDonald, 1999)—had to be reasonably high (i.e., about 0.70 in single-factor models and 0.20 in bifactor models; Gignac & Kretzschmar, 2017).

## Results

For each dataset, we first assessed the model fit of the diffusion model by comparing the actual data with the posterior predictive distribution (i.e., a distribution of simulated data sets, each of the same size as the actual data, generated from the estimated model parameters). Second, we examined the correlation pattern of all parameters, and investigated the relations between them at the latent-variable level through SEM.

### Dataset 1: Young adults from Rey-Mermet et al. (2018)

#### Diffusion modeling

**Fig. 1:**
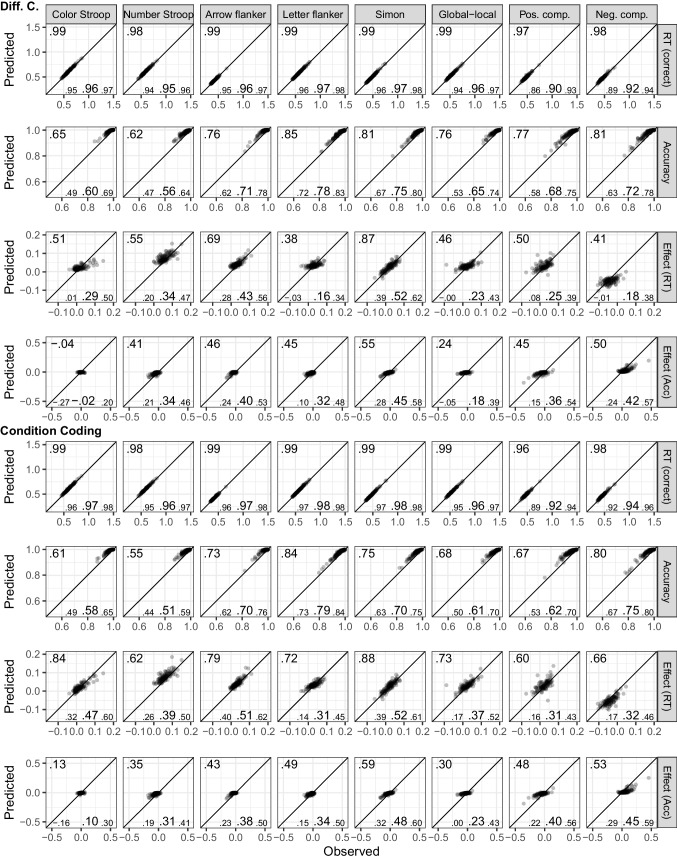
Dataset 1: Young adults from Rey-Mermet et al. (2018). Model fit of diffusion models comparing observed (*x*-axis) with predicted statistics (*y*-axis). First four rows show the fit of the difference-coding parametrization and the last four rows show the fit of the condition-coding parametrization. Each column shows the statistics for one of the eight tasks used in the SEM. The first, second, fifth, and sixth row show overall mean RT (for correct responses) and accuracy across conditions. The third and seventh row, “Effect (RT),” and the fourth and eighth row, “Effect (Acc),” show the congruency effects for RT and accuracy, respectively. Data points are participants and are plotted semitransparently so that overlapping points appear darker. The predicted statistics show the mean of 500 samples from the posterior predictive distribution (i.e., synthetic data of the same size as the observed data generated from the fitted model). The value in the top-left corner of each panel is the concordance correlation coefficient (CCC), which is 1 in case of perfect absolute agreement, for the data points shown. The three values in the lower-left corner represent the 2.5% quantile, the median, and the 97.5% quantile of the CCCs when calculated individually for each sample of the posterior predictive distribution

Figure 1 shows the fits of the diffusion model and includes numerical summaries of fit. We used a measure of absolute agreement between observed data and posterior prediction, the concordance correlation coefficient (*CCC*; Barchard, 2012). For both parametrizations and all tasks, for both posterior predictive mean and across the posterior predictive distribution, mean RTs are recovered almost perfectly ($CCC\ge .97$) and accuracy was recovered reasonably well ($CCC\ge .61$).

Regarding the congruency effects, recovery was worse for the difference coding, which included parameters capturing the congruency effects, than for the condition coding, which did not include such parameters. Furthermore, recovery differed markedly between the posterior predictive mean and across the full posterior predictive distribution. When only considering the former, recovery was good (with few exceptions) for the condition coding. However, recovery across the individual samples of the posterior predictive distribution was in many instances poor, suggesting that the posterior predictive mean paints a rather optimistic picture. The largest misfit was observed for the accuracy congruency effects, where the predicted effects showed a markedly reduced range compared with the observed ones. This suggests that even with a large number of observations, the information provided by the data was sometimes not enough to overwhelm the only weakly informative priors. This particularly applies to the difference coding. Together, because the recovery of the RT congruency effects was still acceptable and only recovery of the accuracy congruency effects was quite weak, individual differences were sufficiently recovered for the present purposes.

#### Correlations and structural equation modeling

Correlations are presented in Table 3 for the drift rate with the difference coding, in Table 4 for the drift rate with the condition coding, in Table 5 for the boundary separation, and in Table 6 for the non-decision time. SEM results are reported next for each of these parameters separately.

**Table 3:** Dataset 1–Young adults from Rey-Mermet et al. (2018): Posterior mean correlation coefficients and 95% credibility intervals for the drift-rate difference estimate of the diffusion model with difference coding (incongruent vs. congruent trials)

Measure	Color Stroop	Number Stroop	Arrow flanker	Letter flanker	Simon	Local	Positive comp
Color Stroop	–						
Number Stroop	− .02 [− .30, .26]	−					
Arrow flanker	− .14 [− .42, .17]	.03 [− .23, .30]	−				
Letter flanker	− .09 [− .37, .21]	.10 [− .18, .37]	.26 [− .01, .51]	−			
Simon	− .13 [− .40, .17]	.04 [− .18, .27]	.08 [− .15, .31]	.21 [− .04, .45]	−		
Local	.11 [− .21, .39]	.03 [− .25, .32]	.06 [− .22, .34]	.09 [− .21, .36]	.19 [− .08, .44]	−	
Positive comp	− .02 [− .31, .27]	.03 [− .22, .28]	.00 [− .25, .25]	.16 [− .11, .41]	.11 [− .11, .33]	.07 [− .21, .35]	−
Negative comp	− .04 [− .32, .23]	.16 [− .10, .41]	.06 [− .20, .33]	.12 [− .16, .37]	.02 [− .21, .25]	.04 [− .23, .30]	.09 [− .15, .35]

Comp. Compatibility. Values in [] are the lower and upper limit of the 95% Bayesian credibility interval (CI). For values printed in bold, the 95% CI excludes 0

**Table 4:** Dataset 1–Young adults from Rey-Mermet et al. (2018): Posterior mean correlation coefficients and 95% credibility intervals for the drift-rates estimates with condition coding

Task	Trial type	Color Stroop		Number Stroop		Arrow flanker		Letter flanker		Simon		Local		Pos comp		Neg comp
		Inc	Con	Inc	Con	Inc	Con	Inc	Con	Inc	Con	Inc	Con	Con	Inc	Inc
Color Stroop	Inc	–														
	Con	**.80** [**.71, .88**]	−													
Number Stroop	Inc	**.21** [**.06, .35**]	**.17** [**.02, .32**]	−												
	Con	**.28** [**.14, .42**]	**.25** [**.10, .39**]	**.61** [**.48, .73**]	−											
Arrow flanker	Inc	**.27** [**.13, .40**]	**.33** [**.19, .46**]	**.23** [**.09, .37**]	**.18** [**.04, .32**]	−										
	Con	**.25** [**.12, .39**]	**.22** [**.08, .35**]	**.27** [**.11, .40**]	**.22** [**.09, .36**]	**.81** [**.72, .88**]	−									
Letter flanker	Inc	.12 [− .03, .27]	.15 [.01, .30]	**.36** [**.21, .50**]	**.31** [**.16, .46**]	**.27** [**.13, .41**]	**.29** [**.15, .42**]	−								
	Con	.11 [− .03, .25]	.09 [− .05, .23]	**.33** [**.19, .47**]	**.30** [**.15, .45**]	**.19** [**.05, .31**]	**.29** [**.16, .40**]	**.71** [**.58, .81**]	−							
Simon	Inc	**.20** [**.05, .34**]	**.23** [**.09, .37**]	**.28** [**.13, .42**]	**.28** [**.13, .43**]	**.17** [**.03, .30**]	**.20** [**.06, .33**]	**.30** [**.15, .44**]	**.33** [**.18, .47**]	−						
	Con	.11 [− .04, .25]	.05 [− .09, .19]	.15 [− .01, .29]	**.18** [**.03, .33**]	.04 [− .09, .18]	.12 [− .02, .26]	**.19** [**.04, .33**]	**.29** [**.14, .43**]	**.65** [**.52, .75**]	−					
Local	Inc	**.36** [**.20, .50**]	**.28** [**.12, .44**]	**.26** [**.10, .42**]	**.27** [**.11, .42**]	**.33** [**.19, .47**]	**.33** [**.18, .46**]	**.27** [**.11, .43**]	**.28** [**.13, .42**]	**.27** [**.11, .42**]	**.24** [**.09, .39**]	−				
	Con	**.23** [**.07, .37**]	**.23** [**.07, .38**]	**.19** [**.03, .35**]	**.21** [**.04, .37**]	**.34** [**.19, .48**]	**.38** [**.23, .51**]	**.30** [**.14, .45**]	**.31** [**.15, .47**]	**.25** [**.08, .40**]	**.34** [**.19, .49**]	**.61** [**.46, .74**]	−			
Pos comp	Inc	.15 [− .01, .30]	**.20** [**.06, .34**]	**.22** [**.07, .38**]	**.25** [**.09, .41**]	**.27** [**.13, .41**]	**.29** [**.14, .43**]	**.26** [**.10, .41**]	**.21** [**.05, .37**]	**.20** [**.05, .35**]	.06 [− .10, .21]	**.21** [**.05, .37]**	.17 [− .00, .33]	−		
	Con	.15 [− .01, .29]	**.19** [**.04, .34**]	.07 [− .09, .24]	.14 [− .03, .30]	**.22** [**.08, .36**]	**.27** [**.12, .40**]	**.25** [**.09, .41**]	**.28** [**.12, .43**]	**.16** [**.00, .32**]	.11 [− .06, .27]	.16 [− .00, .32]	.16 [− .01, .33]	**.52** [**.36, .65**]	−	
Neg. comp	Inc	**.23** [**.08, .37**]	**.25** [**.11, .39**]	.16 [− .00, .31]	**.26** [**.10, .40**]	**.27** [**.13, .40**]	**.26** [**.12, .39**]	**.22** [**.06, .36**]	**.26** [**.09, .40**]	**.26** [**.11, .40**]	.15 [− .01, .29]	**.19** [**.03, .35**]	**.25** [**.10, .40**]	**.35** [**.19, .50**]	**.47** [**.32, .61**]	−
	Con	.12 [− .03, .28]	**.17** [**.03, .33**]	**.21** [**.05, .36**]	**.23** [**.08, .38**]	**.26** [**.12, .39**]	**.26** [**.12, .39**]	**.20** [**.05, .35**]	**.20** [**.05, .36**]	**.28** [**.14, .41**]	.13 [− .02, .29]	**.24** [**.07, .38**]	**.27** [**.11, .42**]	**.31** [**.15, .46**]	**.41** [**.25, .55**]	**.62** [**.48, .74**]

*Inc*. Incongruent, *Con*. Congruent, *Pos. Comp*. Positive compatibility, *Neg. comp*. Negative compatibility. Values in [] are the lower and upper limit of the 95% Bayesian credibility interval (CI). For values printed in bold, the 95% CI excludes 0

**Table 5:** Dataset 1–Young adults from Rey-Mermet et al. (2018): Posterior mean correlation coefficients and 95% credibility intervals for the boundary separation of the diffusion model with condition coding

Measure	Color Stroop	Number Stroop	Arrow flanker	Letter flanker	Simon	Local	Positive comp
Color Stroop	−						
Number Stroop	**.25** **[.10, .40]**	−					
Arrow flanker	**.31** **[.17, .45]**	**.22** **[.08, .38]**	−				
Letter flanker	**.21** **[.06, .36]**	**.32** **[.16, .47]**	**.31** **[.16, .45]**	−			
Simon	**.30** **[.14, .43]**	.13 [− .03, .29]	**.23** **[.08, .37]**	**.40** **[.26, .54]**	−		
Local	**.43** **[.27, .56]**	**.20** **[.05, .36]**	**.36** **[.21, .49]**	**.32** **[.15, .46]**	**.29** **[.13, .44]**	−	
Positive comp	.06 [− .09, .22]	**.29** **[.13, .44]**	.12 [− .04, .28]	**.35** **[.19, .50]**	**.22** **[.06, .37]**	**.16** **[.00, .32]**	−
Negative comp	.09 [− .06, .24]	**.19** **[.03, .35]**	.13 [− .02, .28]	**.15** **[.00, .30]**	.11 [− .04, .27]	.15 [− .01, .30]	**.27** **[.11, .43]**

*Comp*. Compatibility. Values in [] are the lower and upper limit of the 95% Bayesian credibility interval (CI). For values printed in bold, the 95% CI excludes 0

**Table 6:** Dataset 1–Young adults from Rey-Mermet et al. (2018): Posterior mean correlation coefficients and 95% credibility intervals for the non-decision time of the diffusion model with condition coding

Measure	Color Stroop	Number Stroop	Arrow flanker	Letter flanker	Simon	Local	Positive comp
Color Stroop	−						
Number Stroop	**.19** **[.02, .33]**	−					
Arrow flanker	**.19** **[.03, .34]**	.08 [− .08, .22]	−				
Letter flanker	.06 [− .09, .22]	**.40** **[.26, .53]**	**.20** **[.03, .34]**	−			
Simon	**.22** **[.06, .37]**	.16 [− .00, .32]	**.17** **[.01, .32]**	**.34** **[.19, .47]**	−		
Local	**.17** **[.00, .32]**	.10 [− .05, .26]	.14 [− .02, .28]	**.21** **[.05, .35]**	**.23** **[.08, .37]**	−	
Positive comp	.02 [− .14, .18]	.10 [− .05, .25]	**.19** **[.05, .34]**	.16 [− .01, .30]	**.15** **[.00, .30]**	− .01 [− .17, .14]	−
Negative comp	.06 [− .09, .21]	.09 [− .06, .25]	**.22** **[.07, .36]**	.15 [− .00, .30]	.10 [− .07, .26]	− .00 [− .17, .15]	**.35** **[.21, .48]**

*Comp*. Compatibility. Values in [] are the lower and upper limit of the 95% Bayesian credibility interval (CI). For values printed in bold, the 95% CI excludes 0

##### Drift rate with difference coding

 In this analysis, we used the posterior mean correlations of drift-rate differences between congruent and incongruent conditions in each task as the measures of attentional control. Nearly all correlations were low (≤ 0.20), and none were credibly different from zero (see Table 3). Overall, the correlation matrix had a KMO index (0.57) slightly under the lower limit of 0.60, indicating only marginal factorability of the correlation matrix.

**Fig. 2:**
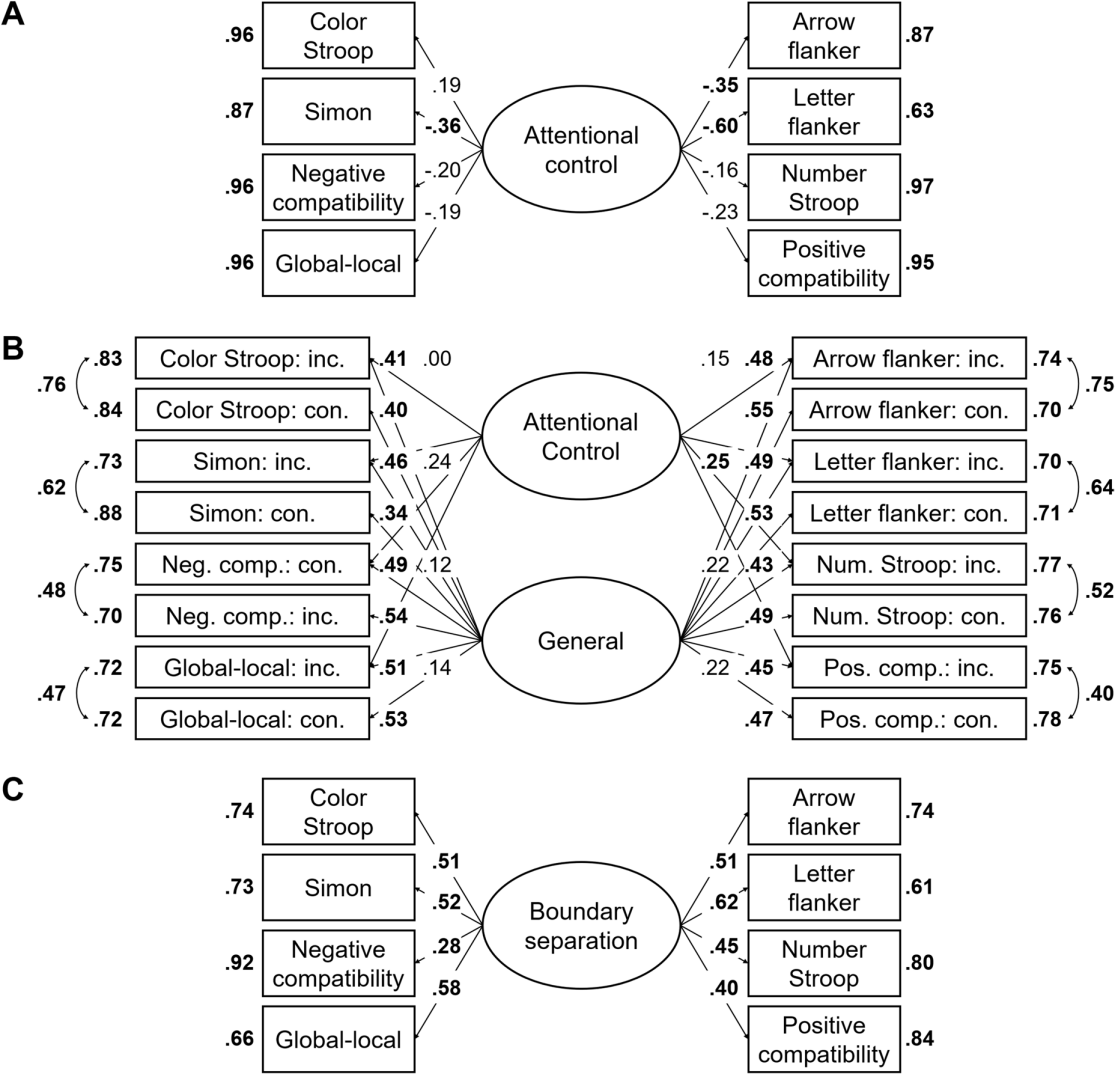
Dataset 1: Young adults from Rey-Mermet et al. (2018). **A** Drift rate with difference coding: One-factor model in which all drift-rate differences between incongruent and congruent trials loaded on a single latent variable (Model 1). **B** Drift rate with condition coding: Bifactor model in which drift rates from the incongruent and congruent trials of all tasks was forced to load on a general factor, and drift rates from the conditions reflecting high attentional control (i.e., the congruent trials of the negative-compatibility task and the incongruent trials of the remaining tasks) were forced to load on an attentional-control factor (Model 2). In this model, factor loadings were constrained to be positive, and error variances from the measures of the same task were allowed to correlate. **C** Boundary separation: One-factor model in which the boundary-separation parameters loaded on a single latent variable (Model 3). For Models 1 and 3, the numbers next to the straight, single-headed arrows are the standardized factor loadings (interpretable as standardized regression coefficients). For Model 2 (bifactor model), for the sake of clarity, these factor loadings are aligned to each measure (i.e., next to the measures for the general factor and next to the factors for the attentional-control factor). For all models, the outward numbers adjacent to each measure are the error variances, attributable to idiosyncratic task requirements and measurement error. The numbers adjacent to the curved, double-headed arrows next to each measure in Model 2 (bifactor model) are the correlations between the error variances. For all values, boldface type indicates *p* < .05. Neg. comp. = Negative compatibility; Pos. comp. = positive compatibility; Num. Stroop = Number Stroop; inc. = incongruent; con. = congruent

In the next step, we aimed to find a coherent factor of attentional control. To this end, we fitted a model in which the drift-rate differences from all tasks loaded on a single factor. This model, referred to as Model 1, is depicted in Fig. 2A. This model provided a good fit to the data, χ^2^(20, *N* = 120) = 11.72, *p* = 0.925, CFI = 1, RMSEA [90% CI] = 0 [0, 0.03], SRMR = 0.05. However, only three measures had loadings larger than 0.30, with the loading of the letter flanker task dominating the loadings. Moreover, error variances were high for most measures (see Fig. 2A), and factor reliability was low (ω = 0.35). Together, this indicates that the factor mainly represents the variance of one measure. Thus, although the model provided a good fit to the data, it had low explanatory power.

##### Drift rate with condition coding

This analysis started from the posterior mean correlations of mean drift rates of congruent and incongruent conditions, and used a bifactor model to extract the variance of attentional control from them. As expected, correlations of drift rates between the incongruent and congruent trials of the same task were high (≥ 0.52) and credible. Furthermore, although all correlations between the different tasks were lower (between 0.04 and 0.41), many were still credible (see Table 4). Overall, the correlation matrix had a good KMO index (0.73).

In the bifactor model, the drift rates of both congruent and incongruent conditions of all tasks were forced to load on a general factor, which reflects variance in all abilities involved in task performance regardless of the condition’s attentional-control demand. The drift rates from the condition assumed to demand more attentional control (i.e., the incongruent condition in all tasks, except for the negative-compatibility task for which the congruent condition demands more control) were forced to additionally load on an attentional-control factor. In this way, the attentional-control factor reflects the common variance across tasks that is not accounted for by the general factor. In addition, error variances from the measures of the same task were allowed to correlate. Moreover, to avoid negative variance, factor loadings were constrained to be positive. This model, thereafter, referred to as Model 2, is depicted in Fig. 2B.

Model 2 provided a good fit to the data, χ^2^(88, *N* = 120) = 86.86, *p* = 0.514, CFI = 1, RMSEA [90% CI] = 0 [0, 0.05], SRMR = 0.06. However, although all measures had loadings larger than 0.34 for the general factor, all loadings for the attentional-control factor were lower than 0.25. Moreover, the factor reliability for this specific factor was very low (ω_h_ = 0.08). Thus, the attentional-control factor did not represent much common variance across the measures.

##### Boundary separation

Most correlations were between 0.20 and 0.43, and credibly differed from zero (see Table 5). Overall, the correlation matrix had a good KMO index (0.76).

In the next step, we fitted a model in which all tasks loaded on a single factor (i.e., Model 3). This model is depicted in Fig. 2C. This model provided an acceptable fit to the data, χ^2^(20, *N* = 120) = 30.44, *p* = 0.063, CFI = 0.91, RMSEA [90% CI] = 0.07 [0, 0.11], SRMR = 0.07. All loadings were larger than 0.40, except for one exception (i.e., the loading of the negative-compatibility task with a value of 0.28). Error variances were low (see Fig. 2C). Factor reliability was also high (ω = 0.70). Thus, these results indicate a coherent factor, reflecting individual differences in speed–accuracy trade-off or caution that generalize across tasks.

##### Non-decision time

Most correlations were low (≤ 0.20) and did not credibly differ from zero (see Table 6). However, the correlation matrix had a good KMO index (0.64).

As before, we fitted a model in which all tasks loaded on a single factor (i.e., Model 4). However, this model provided a bad fit, χ^2^(20, *N* = 120) = 31.95, *p* = 0.044, CFI = 0.82, RMSEA [90% CI] = 0.07 [0.01, 0.11], SRMR = 0.07.

### Dataset 2: Older adults from Rey-Mermet et al. (2018)

#### Diffusion modeling

**Fig. 3:**
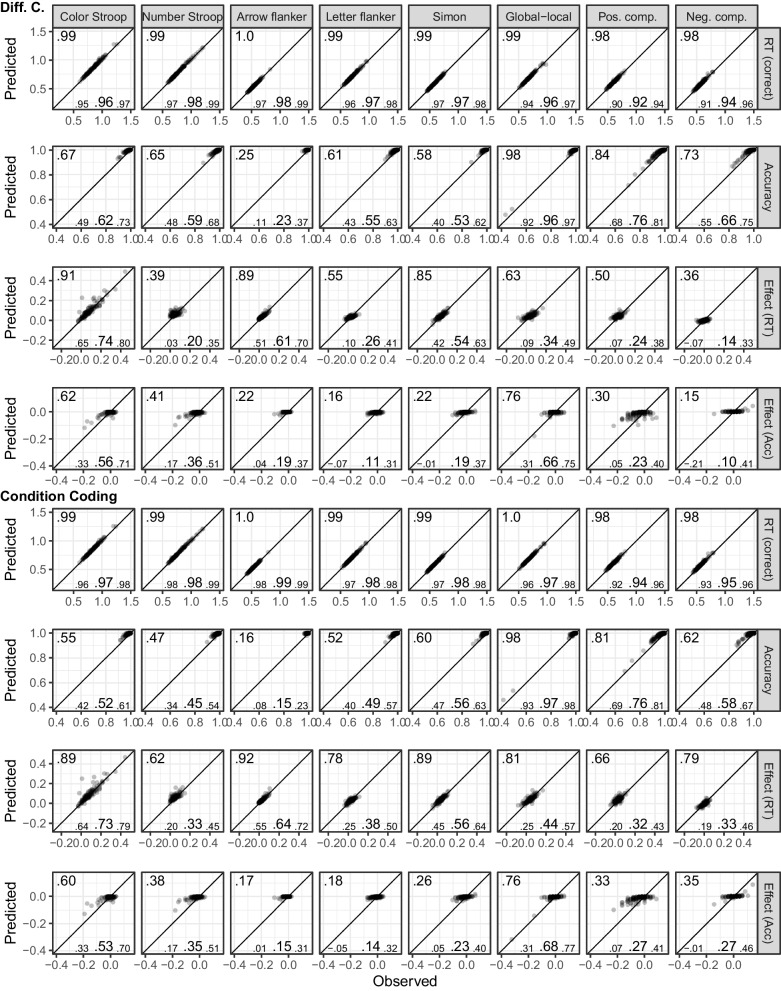
Dataset 2: Older adults from Rey-Mermet et al. (2018). Model fit of diffusion models comparing observed (*x*-axis) with predicted statistics (*y*-axis). See Fig. 1 note for details

Figure 3 shows the fit of the diffusion model for Dataset 2. As for Dataset 1, overall recovery of mean RTs was good ($CCC\ge .98$). For accuracy, visual inspection shows overall good recovery, although *CCC*s are low for cases with ceiling effects and reduced variability. For the congruency effect, we saw a very similar pattern as for Dataset 1. Recovery was better for the condition coding than the difference coding, and the posterior predictive mean recovered markedly better than individual samples from the posterior predictive distribution. One notable difference was that recovery for the RT congruency effect appeared a bit better than for Dataset 1, but recovery of the accuracy effect was worse (i.e., the restricted range in the predicted accuracy effect was even more noticeable). However, overall individual differences were still sufficiently captured.

#### Correlations and structural equation modeling

Correlations are presented in Table 7 for the drift rate with the difference coding, in Table 8 for the drift rate with the condition coding, in Table 9 for the boundary separation, and in Table 10 for the non-decision time. SEM results are reported next for each of these parameters separately.

**Table 7:** Dataset 2–Older adults from Rey-Mermet et al. (2018): Posterior mean correlation coefficients and 95% credibility intervals for the drift-rate difference estimate of the diffusion model with difference coding (incongruent vs. congruent trials)

Measure	Color Stroop	Number Stroop	Arrow flanker	Letter flanker	Simon	Local	Positive comp
Color Stroop	−						
Number Stroop	.11 [− .12, .33]	−					
Arrow flanker	− .06 [− .25, .14]	− .07 [− .29, .17]	−				
Letter flanker	− .06 [− .28, .15]	.09 [− .18, .33]	.22 [− .01, .44]	−			
Simon	− .09 [− .28, .09]	.07 [− .18, .32]	.13 [− .08, .33]	.21 [− .03, .42]	−		
Local	.13 [− .11, .35]	.05 [− .22, .32]	.07 [− .18, .31]	.08 [− .18, .33]	.05 [− .20, .29]	−	
Positive comp	.14 [− .10, .36]	− .01 [− .26, .24]	**.27** **[.03, .50]**	.23 [− .01, .46]	.04 [− .20, .29]	− .06 [− .31, .19]	−
Negative comp	− .14 [− .38, .12]	− .03 [− .31, .25]	− .12 [− .38, .15]	− .08 [− .35, .20]	− .09 [− .33, .18]	.02 [− .26, .30]	− .25 [− .48, .03]

*Comp.* Compatibility. Values in [] are the lower and upper limit of the 95% Bayesian credibility interval (CI). For values printed in bold, the 95% CI excludes 0

**Table 8:** Dataset 2–Older adults from Rey-Mermet et al. (2018): Posterior mean correlation coefficients and 95% credibility intervals for the drift-rates estimates with condition coding

Task	Trial type	Color Stroop		Number Stroop		Arrow flanker		Letter flanker		Simon		Local		Pos comp		Neg comp
		Inc	Con	Inc	Con	Inc	Con	Inc	Con	Inc	Con	Inc	Con	Con	Inc	Inc
Color Stroop	Inc	−														
	Con	**.71** [**.61, .80**]	−													
Number Stroop	Inc	**.20** [**.07, .33**]	**.19** [**.06, .31**]	−												
	Con	.12 [− .01, .25]	**.17** [**.03, .30**]	**.78** [**.69, .86**]	−											
Arrow flanker	Inc	.09 [− .05, .21]	**.25** [**.13, .38**]	.11 [− .02, .25]	**.17** [**.03, .30**]	−										
	Con	.13 [− .01, .26]	**.27** [**.14, .38**]	**.18** [**.05, .30**]	**.20** [**.07, .33**]	**.80** [**.73, .86**]	−									
Letter flanker	Inc	**.17** [**.04, .30**]	**.15** [**.02, .29**]	**.26** [**.13, .39**]	**.24** [**.11, .37**]	**.26** [**.13, .38**]	**.26** [**.13, .40**]	−								
	Con	.12 [− .00, .25]	.08 [− .06, .21]	**.27** [**.14, .40**]	**.26** [**.12, .38**]	**.21** [**.08, .33**]	**.28** [**.15, .41**]	**.81** [**.73, .88**]	−							
Simon	Inc	.01 [− .12, .13]	.10 [− .03, .22]	**.20** [**.07, .32**]	**.28** [**.16, .41**]	**.26** [**.12, .38**]	**.29** [**.16, .41**]	**.41** [**.30, .53**]	**.38** [**.25, .50**]	−						
	Con	− .01 [− .15, .12]	.01 [− .12, .14]	.11 [− .03, .24]	**.19** [**.06, .33**]	**.15** [**.02, .28**]	**.22** [**.09, .35**]	**.30** [**.17, .42**]	**.36** [**.22, .48**]	**.82** [**.74, .88**]	−					
Local	Inc	.05 [− .10, .19]	**.16** [**.02, .30**]	.05 [− .09, .19]	.02 [− .11, .16]	.11 [− .02, .24]	**.18** [**.05, .31**]	.12 [− .02, .25]	.10 [− .05, .23]	**.14** [**.02, .26**]	.10 [− .03, .23]	−				
	Con	.04 [− .11, .18]	**.21** [**.07, .35**]	.07 [− .07, .20]	.04 [− .09, .17]	.13 [− .01, .27]	**.21** [**.08, .33**]	.11 [− .03, .25]	.12 [− .02, .25]	**.20** [**.07, .33**]	**.16** [**.03, .29**]	**.77** [**.67, .85**]	−			
Pos comp	Inc	.13 [− .01, .26]	.04 [− .10, .18]	**.20** [**.07, .34**]	**.23** [**.09, .37**]	**.19** [**.06, .32**]	**.17** [**.04, .30**]	**.17** [**.03, .31**]	**.16** [**.01, .30**]	.14 [.01, .28]	.07 [− .07, .20]	.05 [− .09, .19]	.06 [− .09, .21]	−		
	Con	.06 [− .08, .20]	.05 [− .09, .18]	**.20** [**.06, .34**]	**.24** [**.10, .37**]	**.16** [**.03, .30**]	**.25** [**.11, .37**]	**.16** [**.02, .30**]	**.19** [**.05, .33**]	**.23** [**.09, .35**]	**.15** [**.01, .28**]	**.18** [**.04, .31**]	.11 [− .03, .25]	**.73** [**.62, .82**]	−	
Neg comp	Inc	**.17** [**.04, .29**]	.10 [− .02, .23]	**.26** [**.14, .38**]	**.23** [**.11, .36**]	**.24** [**.12, .36**]	**.27** [**.14, .38**]	**.23** [**.10, .36**]	**.18** [**.06, .30**]	**.20** [**.08, .32**]	.09 [− .04, .22]	.05 [− .08, .18]	**.16** [**.03, .29**]	**.44** [**.31, .56**]	**.43** [**.29, .55**]	−
	Con	**.16** [**.03, .29**]	**.16** [**.04, .29**]	**.20** [**.07, .32**]	**.23** [**.10, .35**]	**.18** [**.06, .29**]	**.25** [**.13, .37**]	**.18** [**.04, .30**]	**.16** [**.04, .29**]	**.18** [**.06, .30**]	.11 [− .01, .23]	.07 [− .06, .20]	**.16** [**.02, .29**]	**.39** [**.25, .52**]	**.48** [**.36, .60**]	**.84** [**.77, .90**]

*Inc*. Incongruent, *Con*. Congruent, *Pos. Comp*. Positive compatibility, *Neg. comp*. Negative compatibility. Values in [] are the lower and upper limit of the 95% Bayesian credibility interval (CI). For values printed in bold, the 95% CI excludes 0

**Table 9:** Dataset 2–Older adults from Rey-Mermet et al. (2018): Posterior mean correlation coefficients and 95% credibility intervals for the boundary separation of the diffusion model with condition coding

Measure	Color Stroop	Number Stroop	Arrow flanker	Letter flanker	Simon	Local	Positive comp
Color Stroop	−						
Number Stroop	**.36** **[.21, .49]**	−					
Arrow flanker	.09 [− .06, .23]	− .06 [− .21, .09]	−				
Letter flanker	**.21** **[.06, .34]**	.08 [− .07, .22]	**.26** **[.11, .40]**	−			
Simon	.01 [− .13, .15]	− .03 [− .18, .12]	**.25** **[.10, .39]**	**.37** **[.23, .49]**	−		
Local	.12 [− .03, .26]	.09 [− .07, .24]	**.18** **[.01, .34]**	**.36** **[.20, .50]**	**.29** **[.13, .44]**	−	
Positive comp	.06 [− .09, .21]	.02 [− .14, .18]	.14 [− .02, .28]	**.21** **[.06, .35]**	**.22** **[.08, .36]**	**.19** **[.02, .36]**	−
Negative comp	**.20** **[.05, .34]**	.12 [− .04, .27]	.11 [− .03, .26]	**.16** **[.02, .31]**	.02 [− .13, .17]	.06 [− .09, .22]	**.20** **[.05, .35]**

*Comp.* Compatibility. Values in [] are the lower and upper limit of the 95% Bayesian credibility interval (CI). For values printed in bold, the 95% CI excludes 0

**Table 10:** Dataset 2–Older adults from Rey-Mermet et al. (2018): Posterior mean correlation coefficients and 95% credibility intervals for the non-decision time of the diffusion model with condition coding

Measure	Color Stroop	Number Stroop	Arrow flanker	Letter flanker	Simon	Local	Positive comp
Color Stroop	−						
Number Stroop	**.26** **[.11, .40]**	−					
Arrow flanker	.13 [− .03, .28]	.11 [− .04, .26]	−				
Letter flanker	**.21** **[.05, .36]**	**.25** **[.09, .39]**	**.33** **[.19, .46]**	−			
Simon	.14 [− .01, .29]	**.22** **[.07, .37]**	**.34** **[.20, .47]**	**.41** **[.26, .53]**	−		
Local	.10 [− .05, .26]	.07 [− .09, .22]	**.26** **[.10, .40]**	**.31** **[.16, .45]**	**.18** **[.03, .32]**	−	
Positive comp	.02 [− .13, .17]	.06 [− .10, .21]	**.17** **[.02, .32]**	.14 [− .02, .28]	**.19** **[.04, .33]**	.10 [− .05, .25]	−
Negative comp	.16 [− .00, .31]	− .01 [− .18, .15]	.11 [− .04, .25]	.13 [− .02, .27]	.14 [− .01, .28]	.05 [− .10, .20]	**.35** **[.21, .48]**

*Comp.* Compatibility. Values in [] are the lower and upper limit of the 95% Bayesian credibility interval (CI). For values printed in bold, the 95% CI excludes 0

##### Drift rate with difference coding

All correlations between drift-rate differences were low (≤ 0.23) and not credible (see Table 7), with one exception (i.e., the correlation between arrow-flanker and positive-compatibility measures). Overall, the correlation matrix had a KMO index (0.56) slightly under the limit of 0.60.

Similar to Dataset 1, we fitted a model in which all drift-rate differences were forced to load on a single factor (i.e., Model 1). This model provided a bad fit to the data, χ^2^(20, *N* = 140) = 24.34, *p* = 0.228, CFI = 0.85, RMSEA [90% CI] = 0.04 [0, 0.09], SRMR = 0.06.

##### Drift rate with condition coding

 Correlations between the drift rates of incongruent and congruent conditions of the same task were high (≥ 0.71) and credible. Furthermore, although all correlations between the different tasks were lower (between 0.04 and 0.48), many were still credible (see Table 8). Overall, the correlation matrix had a good KMO index (0.61).

**Fig. 4:**
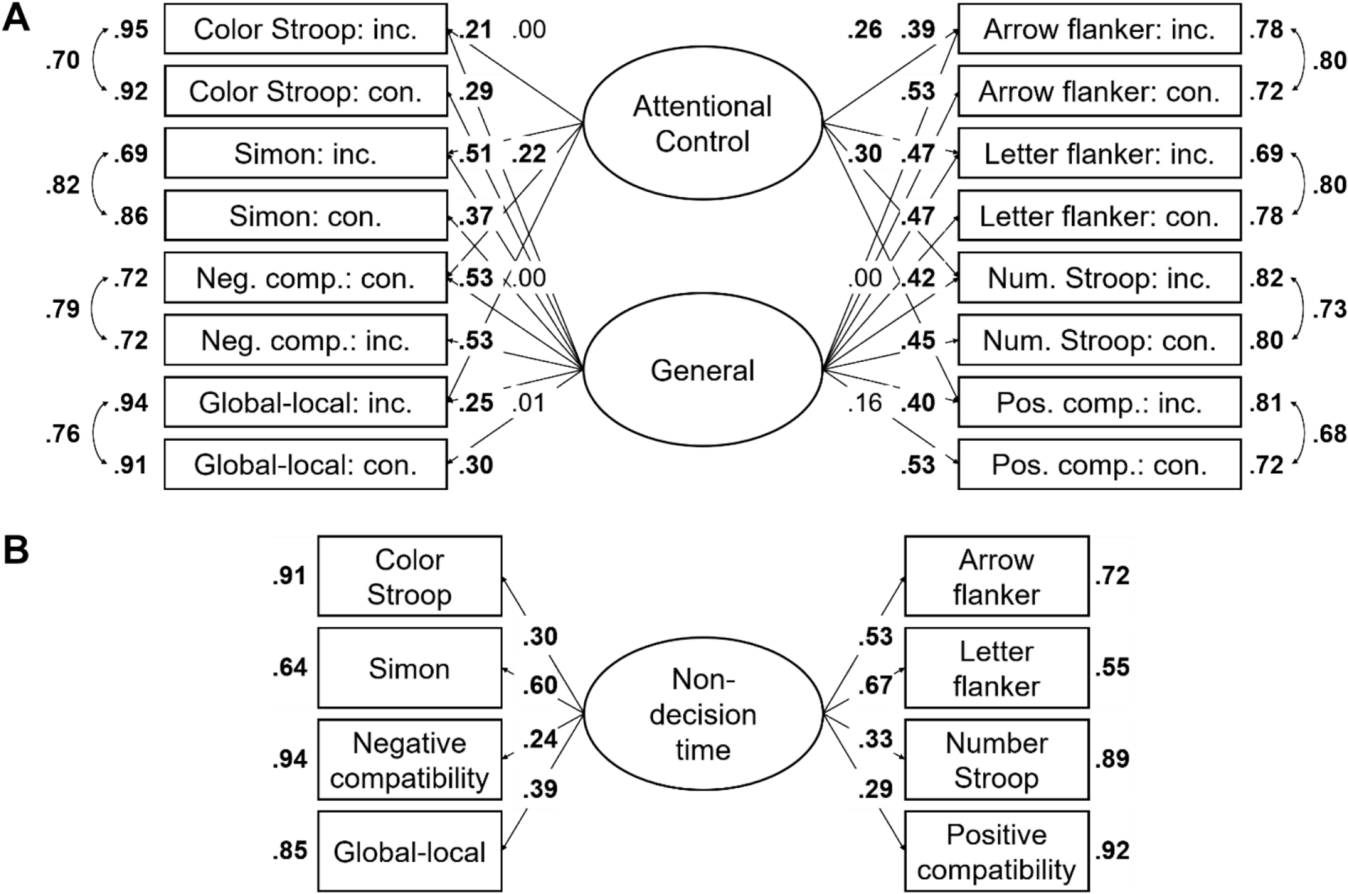
Dataset 2: Older adults from Rey-Mermet et al. (2018). **A** Drift rate with condition coding: Bifactor model in which drift rates from the incongruent and congruent trials of all tasks was forced to load on a general factor, and drift rates from the conditions reflecting high attentional control (i.e., the congruent trials of the negative-compatibility task and the incongruent trials of the remaining tasks) were forced to load on an attentional-control factor (Model 2). In this model, factor loadings were constrained to be positive, and error variances from the measures of the same task were allowed to correlate. **B** Non-decision time: One-factor model in which the non-decision-time parameters loaded on a single latent variable (Model 4). See Fig. 2 note for details

In the next step, we fitted the same bifactor model (Model 2) as for Dataset 1. This model is depicted in Fig. 4A. This model provided a good fit to the data, χ^2^(88, *N* = 140) = 116.21, *p* = 0.024, CFI = 0.98, RMSEA [90% CI] = 0.05 [0.02, 0.07], SRMR = 0.06. Most measures had a loading higher than 0.30 for the general factor. However, all loadings for the attentional-control factor were equal to or lower than 0.30 (see Fig. 4A). Moreover, the reliability for this factor was very low (ω_h_ = 0.05). Thus, this model estimation did not result in a coherent factor of attentional control.

##### Boundary separation

Most correlations were low (≤ 0.20) and not credible (see Table 9). However, the correlation matrix had a good KMO index (0.68).

Similar to Dataset 1, we fitted a single-factor model (i.e., Model 3). However, this model provided a bad fit, χ^2^(20, *N* = 140) = 33.44, *p* = 0.030, CFI = 0.84, RMSEA [90% CI] = 0.07 [0.02, 0.11], SRMR = 0.08.

##### Non-decision time

 Most correlations were low (≤ 0.20) and not credible (see Table 10). Overall, the correlation matrix had a good KMO index (0.70).

As before, we fitted a single-factor model (i.e., Model 4). This model is depicted in Fig. 4B. This model provided an acceptable fit to the data, χ^2^(20, *N* = 140) = 28.69, *p* = 0.094, CFI = 0.91, RMSEA [90% CI] = 0.06 [0, 0.10], SRMR = 0.06. All loadings ranged from 0.24 to 0.67, and error variances were moderate (see Fig. 4B). Factor reliability was only of modest size (ω = 0.56). Thus, the results suggest a moderately coherent factor.

### Dataset 3: Experiment 1 from Whitehead et al. (2019)

#### Diffusion modeling

**Fig. 5:**
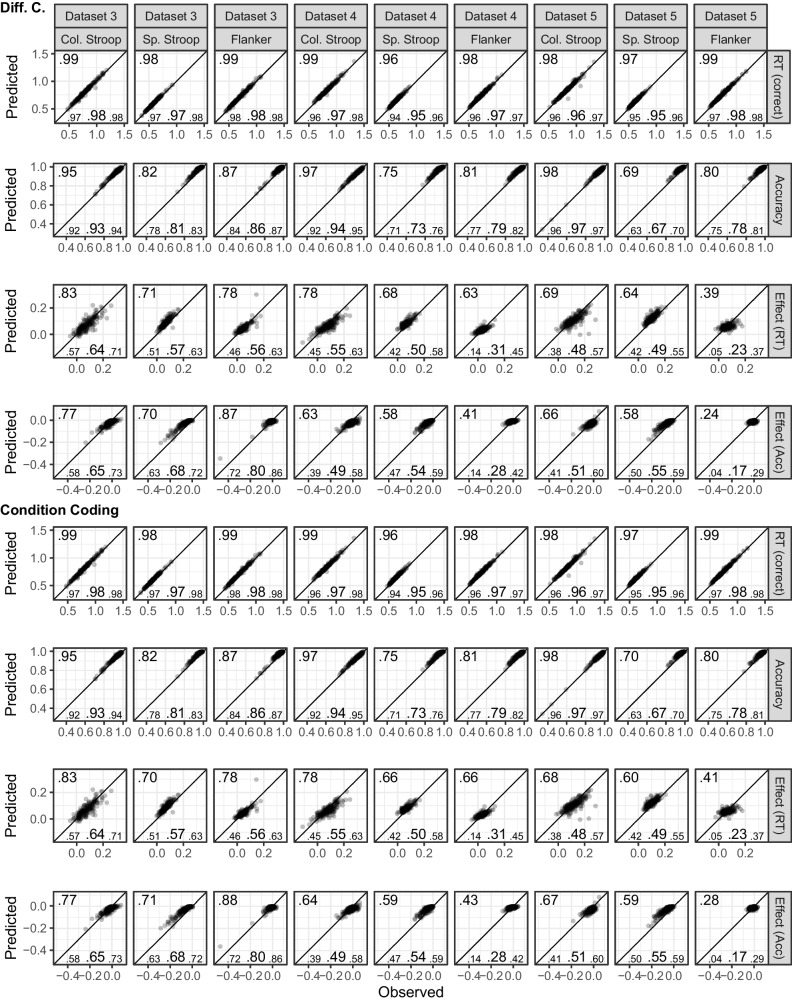
Datasets 3, 4, and 5—Experiments 1, 2, and 3, respectively, from Whitehead et al. (2019): Model fit of diffusion models comparing observed (*x*-axis) with predicted statistics (*y*-axis). See Fig. 1 note for details

Figure 5 (Columns 1 to 3) shows the fit of the diffusion model. As before, recovery of the response times and accuracy was very good ($CCC\ge .98 \text{ and } CCC\ge .82,\text{ respectively}$). In addition, the recovery of the congruency effects both for response times ($CCC\ge .70$) and accuracy ($CCC\ge .70$) was very good. Finally, the overall very positive recovery of individual differences holds for both posterior predictive means and also generally across the individual samples of the posterior predictive distributions. Thus, fit is noticeably better in Dataset 3 than in Datasets 1 and 2.

#### Correlations and structural equation modeling

Correlations are presented in the upper part of Table 11 for the drift rate with the difference coding, in the upper part of Table 12 for the drift rate with the condition coding, in the upper part of Table 13 for the boundary separation, and in the upper part of Table 14 for the non-decision time. SEM results are reported next for each of these parameters separately.

**Table 11:** Datasets 3, 4, and 5–Experiments 1, 2, and 3, respectively, from Whitehead et al. (2019): Posterior mean correlation coefficients and 95% credibility intervals for the drift-rate difference estimate of the diffusion model with difference coding (incongruent vs. congruent trials)

Dataset	Measure	Color Stroop	Spatial Stroop
3	Color Stroop	−	
	Spatial Stroop	.14 [− .04, .31]	−
	Flanker	.08 [− .10, .26]	− .02 [− .20, .15]
4	Color Stroop	−	
	Spatial Stroop	**.20** **[.01, .39]**	−
	Flanker	.14 [− .10, .38]	**.31** **[.08, .52]**
5	Color Stroop	−	
	Spatial Stroop	**.17** **[.01, .32]**	−
	Flanker	**.26** **[.07, .46]**	**.30** **[.10, .47]**

Values in [] are the lower and upper limit of the 95% Bayesian credibility interval (CI). For values printed in bold, the 95% CI excludes 0

**Table 12:** Datasets 3, 4, and 5–Experiments 1, 2, and 3, respectively, from Whitehead et al. (2019): Posterior mean correlation coefficients and 95% credibility intervals for the drift-rates estimates with condition coding

Dataset	Task	Trial type	Color Stroop		Spatial Stroop		Flanker
			Inc	Con	Inc	Con	Inc
3	Color Stroop	Inc	−				
		Con	**.86** **[.81, .90]**	−			
	Spatial Stroop	Inc	**.42** **[.30, .54]**	**.40** **[.27, .51]**	−		
		Con	**.37** **[.24, .49]**	**.38** **[.25, .49]**	**.88** **[.84, .92]**	−	
	Flanker	Inc	**.33** **[.20, .45]**	**.29** **[.14, .41]**	**.32** **[.20, .45]**	**.38** **[.26, .50]**	−
		Con	**.33** **[.21, .46]**	**.31** **[.16, .43]**	**.34** **[.21, .47]**	**.38** **[.26, .51]**	**.94** **[.91, .96]**
4	Color Stroop	Inc	−				
		Con	**.88** **[.84, .91]**	−			
	Spatial Stroop	Inc	**.45** **[.34, .56]**	**.39** **[.26, .50]**	−		
		Con	**.40** **[.27, .50]**	**.37** **[.25, .49]**	**.93** **[.91, .95]**	−	
	Flanker	Inc	**.41** **[.29, .52]**	**.37** **[.24, .49]**	**.47** **[.36, .57]**	**.46** **[.35, .56]**	−
		Con	**.40** **[.28, .52]**	**.38** **[.25, .50]**	**.46** **[.35, .57]**	**.47** **[.36, .58]**	**.97** **[.96, .98]**
5	Color Stroop	Inc	−				
		Con	**.92** **[.89, .94]**	−			
	Spatial Stroop	Inc	**.36** **[.24, .48]**	**.25** **[.12, .37]**	−		
		Con	**.30** **[.18, .42]**	**.25** **[.12, .36]**	**.83** **[.78, .88]**	−	
	Flanker	Inc	**.32** **[.19, .44]**	**.25** **[.12, .37]**	**.44** **[.33, .55]**	**.46** **[.34, .56]**	−
		Con	**.34** **[.21, .45]**	**.29** **[.16, .41]**	**.40** **[.28, .51]**	**.44** **[.32, .54]**	**.99** **[.98, .99]**

*Inc*. Incongruent, *Con*. Congruent. Values in [] are the lower and upper limit of the 95% Bayesian credibility interval (CI). For values printed in bold, the 95% CI excludes 0

**Table 13:** Datasets 3, 4, and 5–Experiments 1, 2, and 3, respectively, from Whitehead et al. (2019): Posterior mean correlation coefficients and 95% credibility intervals for the boundary separation of the diffusion model with condition coding

Dataset	Measure	Color Stroop	Spatial Stroop
3	Color Stroop	−	
	Spatial Stroop	**.37** **[.24, .50]**	−
	Flanker	**.46** **[.34, .57]**	**.41** **[.29, .53]**
4	Color Stroop	−	
	Spatial Stroop	**.53** **[.42, .63]**	−
	Flanker	**.50** **[.38, .60]**	**.49** **[.37, .60]**
5	Color Stroop	−	
	Spatial Stroop	**.51** **[.40, .61]**	−
	Flanker	**.56** **[.46, .65]**	**.60** **[.50, .69]**

Values in [] are the lower and upper limit of the 95% Bayesian credibility interval (CI). For values printed in bold, the 95% CI excludes 0

**Table 14:** Datasets 3, 4, and 5–Experiments 1, 2, and 3, respectively, from Whitehead et al. (2019): Posterior mean correlation coefficients and 95% credibility intervals for the non-decision time of the diffusion model with condition coding

Dataset	Measure	Color Stroop	Spatial Stroop
3	Color Stroop	−	
	Spatial Stroop	**.33** **[.20, .46]**	−
	Flanker	**.43** **[.31, .55]**	**.37** **[.24, .49]**
4	Color Stroop	−	
	Spatial Stroop	**.26** **[.11, .38]**	−
	Flanker	**.37** **[.24, .49]**	**.23** **[.10, .35]**
5	Color Stroop	−	
	Spatial Stroop	**.29** **[.16, .41]**	−
	Flanker	**.49** **[.37, .59]**	**.38** **[.26, .49]**

Values in [] are the lower and upper limit of the 95% Bayesian credibility interval (CI). For values printed in bold, the 95% CI excludes 0

##### Drift rate with difference coding

All correlations between drift-rate differences were low (≤ 0.14) and not credible (see Table 11, upper part). Overall, the correlation matrix had a low KMO index (0.48).

The next step was to find a coherent factor of attentional control using the three attentional-control measures. To this end, we fitted a saturated model in which the drift-rate differences from all three tasks loaded on a single factor (i.e., Model 5). However, in line with the low KMO index, this model did not converge for Dataset 3.

##### Drift rate with condition coding

Correlations between the drift rates of incongruent and congruent conditions of the same task were high (≥ 0.86) and credible. Furthermore, although all correlations between the different tasks were lower, they were still moderate (between 0.29 and 0.42) and credible (see Table 12, upper part). Overall, the correlation matrix had a good KMO index (0.61).

In the next step, we aimed to establish a coherent factor of attentional control using a bifactor-modeling approach. Accordingly, in Model 6, the drift rates of both congruent and incongruent conditions of all three tasks were forced to load on a general factor, and the drift rates of incongruent trials were also forced to load on an attentional-control factor. In addition, in line with the previous computations of the bifactor models in the present study, error variances from the measures of the same task were allowed to correlate, and factor loadings were constrained to be positive. However, the SEM estimation showed that the standard errors could not be computed, and some estimated observed variances were negative, suggesting that Model 6 was not identified.

##### Boundary separation

All correlations were moderate (between 0.37 and 0.46) and credible (see Table 13, upper part). Furthermore, the correlation matrix had a good KMO index (0.66).

**Fig. 6:**
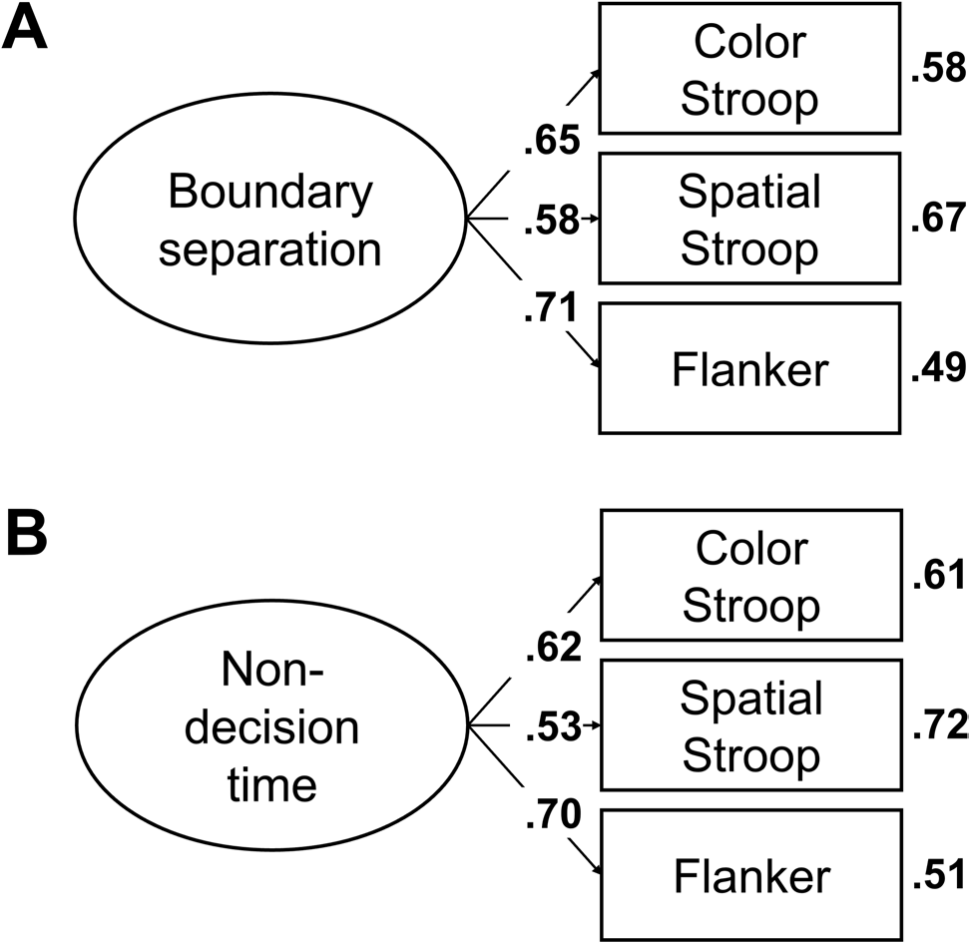
Dataset 3: Experiment 1 from Whitehead et al. (2019). **A** Boundary separation: One-factor model in which the boundary-separation parameters loaded on a single latent variable (Model 7). **B** Non-decision time: One-factor model in which the non-decision-time parameters loaded on a single latent variable (Model 8). See Fig. 2 note for details

In the next step, we fitted a model in which all tasks loaded on a single factor (i.e., Model 7). This model is depicted in Fig. 6A. This model converged, and as it is saturated, it provided a perfect fit to the data. All loadings were relatively high (i.e., larger than 0.58), and error variances were relatively low (see Fig. 6A). Factor reliability was quite high (ω = 0.69). Thus, these results support the view of a coherent factor reflecting individual differences in speed–accuracy trade-off or caution that generalize across tasks.

##### Non-decision time

All correlations were moderate (between 0.33 and 0.43) and credible (see Table 14, upper part). Overall, the correlation matrix had a good KMO index (0.65).

As before, we fitted a model in which all tasks loaded on a single factor (i.e., Model 8). This model is depicted in Fig. 6B. As this model is saturated, it provided a perfect fit to the data. All loadings were relatively high (i.e., larger than 0.53), and error variances were relatively low (see Fig. 6B). Factor reliability was quite high (ω = 0.66). Thus, the results suggest a coherent factor for the non-decision time.

### Dataset 4: Experiment 2 from Whitehead et al. (2019)

#### Diffusion modeling

Figure 5 (Columns 4 to 6) shows the fit of the diffusion model. Recovery was very good for response times ($CCC\ge .96$) and accuracy ($CCC\ge .75$). For congruency effects, recovery was also good. The only exception was the accuracy congruency effect for the flanker task with $CCC\approx .40$ (all other $CCC\ge .58$). Overall, this indicates a good fit for the posterior predictive means. The same result was observed for the individual samples from the posterior predictive distribution. The only exception was that the RT and accuracy congruency effects of the flanker task showed lower *CCC*s. Therefore, these results were similar to Dataset 3 and showed that individual differences were well recovered.

#### Correlations and structural equation modeling

Correlations are presented in the middle part of Table 11 for the drift rate with the difference coding, in the middle part of Table 12 for the drift rate with the condition coding, in the middle part of Table 13 for the boundary separation, and in the middle part of Table 14 for the non-decision time. SEM results are reported next for each of these parameters separately.

##### Drift rate with difference coding

All correlations between drift-rate differences were of modest size (≤ 0.31), but only one correlation was not credible (see Table 11, middle part). Overall, the correlation matrix had a KMO index (0.57) slightly under the lower limit of 0.60, indicating only marginal factorability of the correlation matrix.

**Fig. 7:**
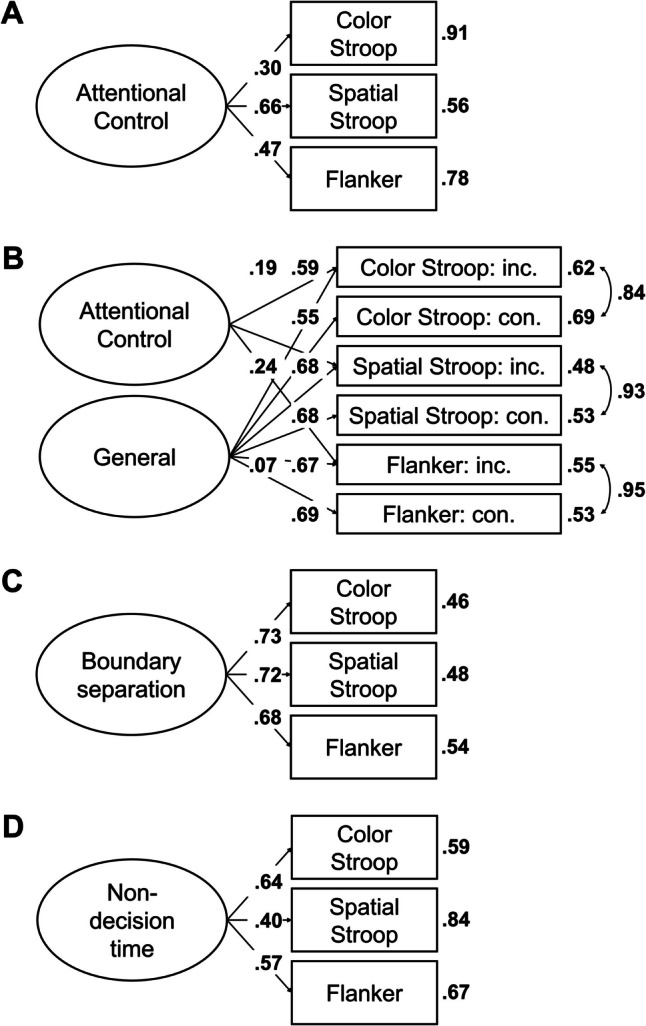
Dataset 4: Experiment 2 from Whitehead et al. (2019). **A** Drift rate with difference coding: One-factor model in which all drift-rate differences between incongruent and congruent trials loaded on a single latent variable (Model 5). **B** Drift rate with condition coding: Bifactor model in which drift rates from the incongruent and congruent trials of all tasks was forced to load on a general factor, and drift rates from the incongruent trials were forced to load on an attentional-control factor (Model 6). In this model, factor loadings were constrained to be positive, and error variances from the measures of the same task were allowed to correlate. **C** Boundary separation: One-factor model in which the boundary-separation parameters loaded on a single latent variable (Model 7). **D** Non-decision time: One-factor model in which the non-decision-time parameters loaded on a single latent variable (Model 8). See Fig. 2 note for details

Similar to Dataset 3, we fitted a model in which the drift-rate differences from all three tasks loaded on a single factor (i.e., Model 5). This model is depicted in Fig. 7. As this model is saturated, it provided a perfect fit to the data. All loadings were larger than 0.30, with the loading of the spatial Stroop task being higher than the other loadings. Error variances were moderate, and factor reliability was only of modest size (ω = 0.51). Together, these results suggest a low to moderately coherent factor at best.

##### Drift rate with condition coding

 Correlations between the drift rates of incongruent and congruent conditions of the same task were high (≥ 0.88) and credible. Furthermore, although all correlations between the different tasks were lower, they were still moderate (between 0.37 and 0.47) and credible (see Table 12, middle part). Overall, the correlation matrix had a good KMO index (0.62).

In the next step, we fitted the same bifactor model (Model 6) as for Dataset 3. This model is depicted in Fig. 7B. This model provided a good fit to the data, χ^2^(3, *N* = 195) = 0.78, *p* = 0.854, CFI = 1, RMSEA [90% CI] = 0 [0, 0.07], SRMR = 0. All loadings for the general factor were relatively high (i.e., larger than 0.55). However, all loadings for the attentional-control factor were low (≤ 0.24, see Fig. 7B). Moreover, the reliability for this specific factor was very low (ω_h_ = 0.04). Thus, this model estimation did not result in a coherent factor of attentional control.

##### Boundary separation 

All correlations were quite high (between 0.49 and 0.53) and credible (see Table 13, middle part). Furthermore, the correlation matrix had a good KMO index (0.69).

Similar to Dataset 3, we fitted the model in which all tasks loaded on a single factor (i.e., Model 7). This model is depicted in Fig. 7C. As this model is saturated, it provided a perfect fit to the data. All loadings were high (i.e., larger than 0.68), and error variances were low (see Fig. 7C). Factor reliability was high (ω = 0.75). Thus, these results are again in line with a coherent factor representing individual differences in speed–accuracy trade-off or caution that generalize across tasks.

##### Non-decision time

 The correlations were low to moderate (between 0.23 and 0.37) and credible (see Table 14, middle part). Overall, the correlation matrix had an acceptable KMO index (0.60).

As before, we fitted a model in which all tasks loaded on one factor (i.e., Model 8). This model is depicted in Fig. 7D. As this model is saturated, it provided a perfect fit to the data. All loadings were larger than 0.40. However, error variances were moderate (see Fig. 7D), and factor reliability was only of modest size (ω = 0.56). Thus, the results suggest a moderately coherent factor.

### Dataset 5: Experiment 3 from Whitehead et al. (2019)

#### Diffusion modeling

Figure 5 (Columns 7 to 9) shows the fit of the diffusion model. Recovery was very good for response times ($CCC\ge .97$) and not much worse for accuracy ($CCC\ge .69$). For congruency effects, recovery was good for the color and spatial Stroop tasks ($CCC\ge .58$), but less good for the flanker task ($CCC\ge .24$). Thus, for the posterior predictive means, recovery was good in all measures with the exception of the flanker congruency effects. The same pattern was observed for individual samples from the posterior predictive distribution. Therefore, these results were similar to Datasets 3 and 4 and showed that individual differences were overall well recovered.

#### Correlations and structural equation modeling

Correlations are presented in the lower part of Table 11 for the drift rate with the difference coding, in the lower part of Table 12 for the drift rate with the condition coding, in the lower part of Table 13 for the boundary separation, and in the lower part of Table 14 for the non-decision time. SEM results are reported next for each of these parameters separately.

##### Drift rate with difference coding

All correlations between drift-rate differences were of modest size (≤ 0.30) but credible (see Table 11, lower part). Overall, the correlation matrix had a KMO index (0.59) slightly under the lower limit of 0.60, indicating only marginal factorability of the correlation matrix.

**Fig. 8:**
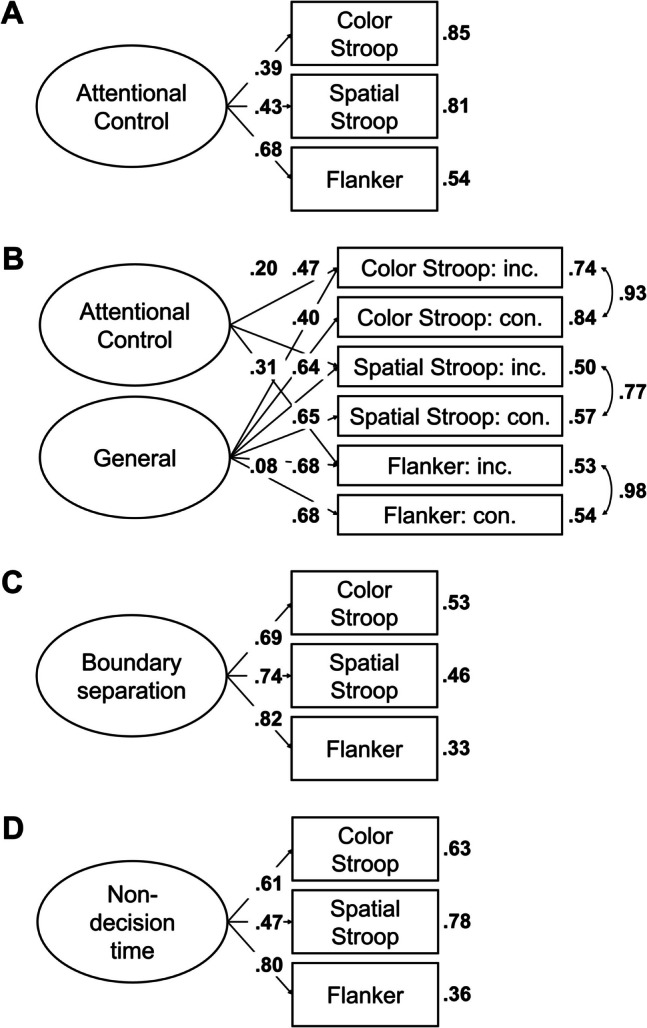
Dataset 5: Experiment 3 from Whitehead et al. (2019). **A** Drift rate with difference coding: One-factor model in which all drift-rate differences between incongruent and congruent trials loaded on a single latent variable (Model 5). **B** Drift rate with condition coding: Bifactor model in which drift rates from the incongruent and congruent trials of all tasks was forced to load on a general factor, and drift rates from the incongruent trials were forced to load on an attentional-control factor (Model 6). In this model, factor loadings were constrained to be positive, and error variances from the measures of the same task were allowed to correlate. **C** Boundary separation: One-factor model in which the boundary-separation parameters loaded on a single latent variable (Model 7). **D** Non-decision time: One-factor model in which the non-decision-time parameters loaded on a single latent variable (Model 8). See Fig. 2 note for details

Similar to Datasets 3 and 4, we fitted the model in which the drift-rate differences from all three tasks loaded on a single factor (i.e., Model 5). This model is depicted in Fig. 8A. As this model is saturated, it provided a perfect fit to the data. All loadings were larger than 0.30, with the loading of the flanker task dominating the remaining loadings (see Fig. 8A). Error variances were moderate, and factor reliability was low (ω = 0.41). Together, this indicates that the factor mainly represents the variance of the flanker measure, suggesting that the model had low explanatory power.

##### Drift rate with condition coding 

Correlations between the drift rates of incongruent and congruent conditions of the same task were high (≥ 0.83) and credible. Furthermore, the correlations between the different tasks were lower, ranging from low to moderate (between 0.25 and 0.46), but all were still credible (see Table 12, lower part). Overall, the KMO index for the correlation matrix (0.57) is slightly under the lower limit of 0.60, indicating only marginal factorability of the correlation matrix.

In the next step, we fitted the same bifactor model (Model 6) as for Datasets 3 and 4. This model is depicted in Fig. 8B. This model provided a good fit to the data, χ^2^(3, *N* = 210) = 25.70, *p* < 0.001, CFI = 0.99, RMSEA [90% CI] = 0.19 [0.13, 0.26], SRMR = 0.02. All loadings for the general factor were larger than 0.40. However, all loadings for the attentional-control factor were low (≤ 0.31), with the loading of the spatial Stroop task being higher than the other loadings (see Fig. 8B). Moreover, the reliability for this specific factor was very low (ω_h_ = 0.07). Thus, this model estimation did not result in a coherent factor of attentional control.

##### Boundary separation

All correlations were quite high (between 0.51 and 0.60) and credible (see Table 13, lower part). Furthermore, the correlation matrix had a good KMO index (0.70).

Similar to Datasets 3 and 4, we fitted the model in which all tasks loaded on one factor (i.e., Model 7). This model is depicted in Fig. 8C. As it saturated, it provided a perfect fit to the data. All loadings were high (i.e., larger than 0.69), and error variances were low (see Fig. 8C). Factor reliability was high (ω = 0.80). Thus, these results show a coherent factor reflecting individual differences in speed–accuracy trade-off or caution that generalize across tasks.

##### Non-decision time 

The correlations were moderate (between 0.29 and 0.49) and credible (see Table 14, lower part). Overall, the correlation matrix had a good KMO index (0.62).

As before, we fitted a model in which all tasks loaded on a single factor (Model 8). This model is depicted in Fig. 8D. As this model is saturated, it provided a perfect fit to the data. All tasks were larger than 0.47, and error variances were moderate (see Fig. 8D). Factor reliability was slightly under the lower limit of 0.70 (ω = 0.69). Together, the results suggest a coherent factor.

### Dataset 6: Kane et al. (2016)

#### Diffusion modeling

**Fig. 9:**
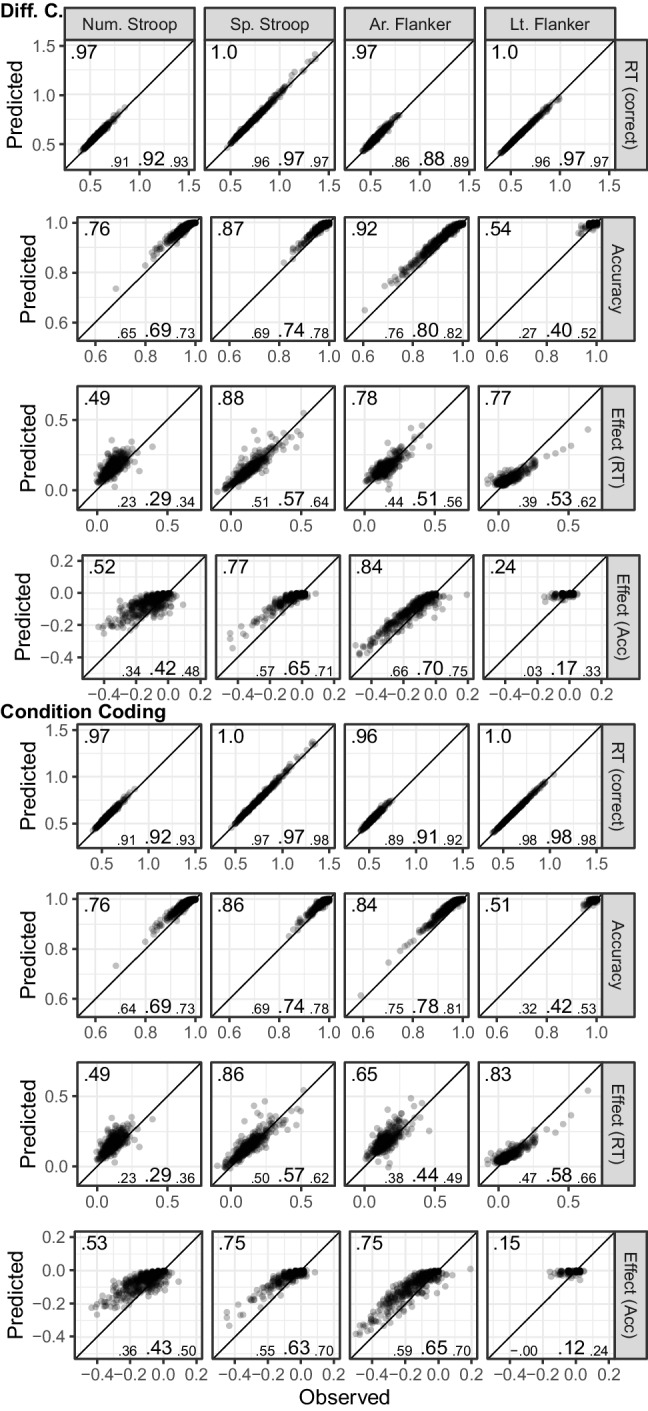
Dataset 6: Kane et al. (2016). Model fit of diffusion models comparing observed (*x*-axis) with predicted statistics (*y*-axis). See Fig. 1 note for details

Figure 9 shows the fit of the diffusion model. For the spatial Stroop and arrow flanker tasks recovery was as before very good for both response times and accuracy ($CCC\ge .96 \text{ and } CCC\ge .84, \text{ respectively}$) as well as the associated congruency effects ($CCC\ge .65 \text{ and } CCC\ge .75, \text{ respectively}$). For the number Stroop task, the recovery was similar for mean response time and accuracy, but recovery of congruency effects was somewhat worse ($CCC\approx .5$). For the letter flanker task, we again saw good recovery for response times ($CCC=1$) and associated congruency effects ($CCC\ge .77$), but recovery of accuracy and associated congruency effects was low due to a reduced range (i.e., a ceiling effect). The pattern for the individual samples from the posterior distribution was also very similar, with the only cases showing poor recovery being the ones mentioned before. Overall, fit is similar to the fit of the previous datasets—somewhat worse than for Datasets 3 to 5, but better than for Datasets 1 and 2—and recovers individual differences reasonably well, if such differences were present (i.e., not for accuracy of the letter flanker task).

#### Correlations and structural equation modeling

Correlations are presented in Table 15 for the drift rate with the difference coding, in Table 16 for the drift rate with the condition coding, in Table 17 for the boundary separation, and in Table 18 for the non-decision time. SEM results are reported next for each of these parameters separately.

**Table 15:** Dataset 6 from Kane et al. (2016): Posterior mean correlation coefficients and 95% credibility intervals for the drift-rate difference estimate of the diffusion model with difference coding (incongruent vs. congruent trials)

Measure	Number Stroop	Spatial Stroop	Arrow flanker
Number Stroop	−		
Spatial Stroop	.15 [− .02, .30]	−	
Arrow flanker	**.25** **[.10, .39]**	.04 [− .10, .17]	−
Letter flanker	**.25** **[.04, .46]**	.00 [− .20, .20]	**.32** **[.13, .50]**

Values in [] are the lower and upper limit of the 95% Bayesian credibility interval (CI). For values printed in bold, the 95% CI excludes 0

**Table 16:** Dataset 6 from Kane et al. (2016): Posterior mean correlation coefficients and 95% credibility intervals for the drift-rates estimates with condition coding

Task	Trial type	Number Stroop		Spatial Stroop		Arrow flanker		Letter flanker
		Inc	Con	Inc	Con	Inc	Con	Inc
Number Stroop	Inc	−						
	Con	**.77** **[.71, .83]**	−					
Spatial Stroop	Inc	**.15** **[.04, .26]**	**.25** **[.16, .35]**	−				
	Con	.09 [− .02, .20]	**.24** **[.14, .33]**	**.74** **[.68, .79]**	−			
Arrow flanker	Inc	**.45** **[.35, .55]**	**.37** **[.27, .46]**	**.14** **[.02, .24]**	.07 [− .03, .18]	−		
	Con	**.32** **[.22, .43]**	**.39** **[.30, .48]**	**.27** **[.17, .37]**	**.23** **[.12, .33]**	**.52** **[.43, .60]**	** − **	
Letter flanker	Inc	**.23** **[.12, .34]**	**.31** **[.21, .41]**	**.24** **[.12, .35]**	**.17** **[.07, .27]**	**.20** **[.09, .30]**	**.30** **[.20, .40]**	−
	Con	**.20** **[.10, .31]**	**.32** **[.23, .41]**	**.26** **[.15, .36]**	**.20** **[.10, .29]**	**.13** **[.03, .24]**	**.34** **[.34, .43]**	**.90** **[.86, .93]**

*Inc*. Incongruent, *Con*. Congruent. Values in [] are the lower and upper limit of the 95% Bayesian credibility interval (CI). For values printed in bold, the 95% CI excludes 0

**Table 17:** Dataset 6 from Kane et al. (2016): Posterior mean correlation coefficients and 95% credibility intervals for the boundary separation of the diffusion model with condition coding

Measure	Number Stroop	Spatial Stroop	Arrow flanker
Number Stroop	** − **		
Spatial Stroop	**.42** **[.31, .51]**	** − **	
Arrow flanker	**.63** **[.55, .70]**	**.37** **[.25, .47]**	** − **
Letter flanker	**.32** **[.21, .43]**	**.32** **[.20, .43]**	**.43** **[.32, .53]**

Values in [] are the lower and upper limit of the 95% Bayesian credibility interval (CI). For values printed in bold, the 95% CI excludes 0

**Table 18:** Dataset 6 from Kane et al. (2016): Posterior mean correlation coefficients and 95% credibility intervals for the non-decision time of the diffusion model with condition coding

Measure	Number Stroop	Spatial Stroop	Arrow flanker
Number Stroop	−		
Spatial Stroop	**.23** **[.12, .32]**	** − **	
Arrow flanker	**.41** **[.32, .50]**	**.29** **[.19, .39]**	** − **
Letter flanker	**.19** **[.08, .29]**	**.26** **[.15, .36]**	**.14** **[.02, .25]**

Values in [] are the lower and upper limit of the 95% Bayesian credibility interval (CI). For values printed in bold, the 95% CI excludes 0

##### Drift rate with difference coding 

All correlations between drift-rate differences were of modest size (≤ 0.32) and only half were credible (see Table 15). Overall, the correlation matrix had a KMO index of 0.60.

**Fig. 10:**
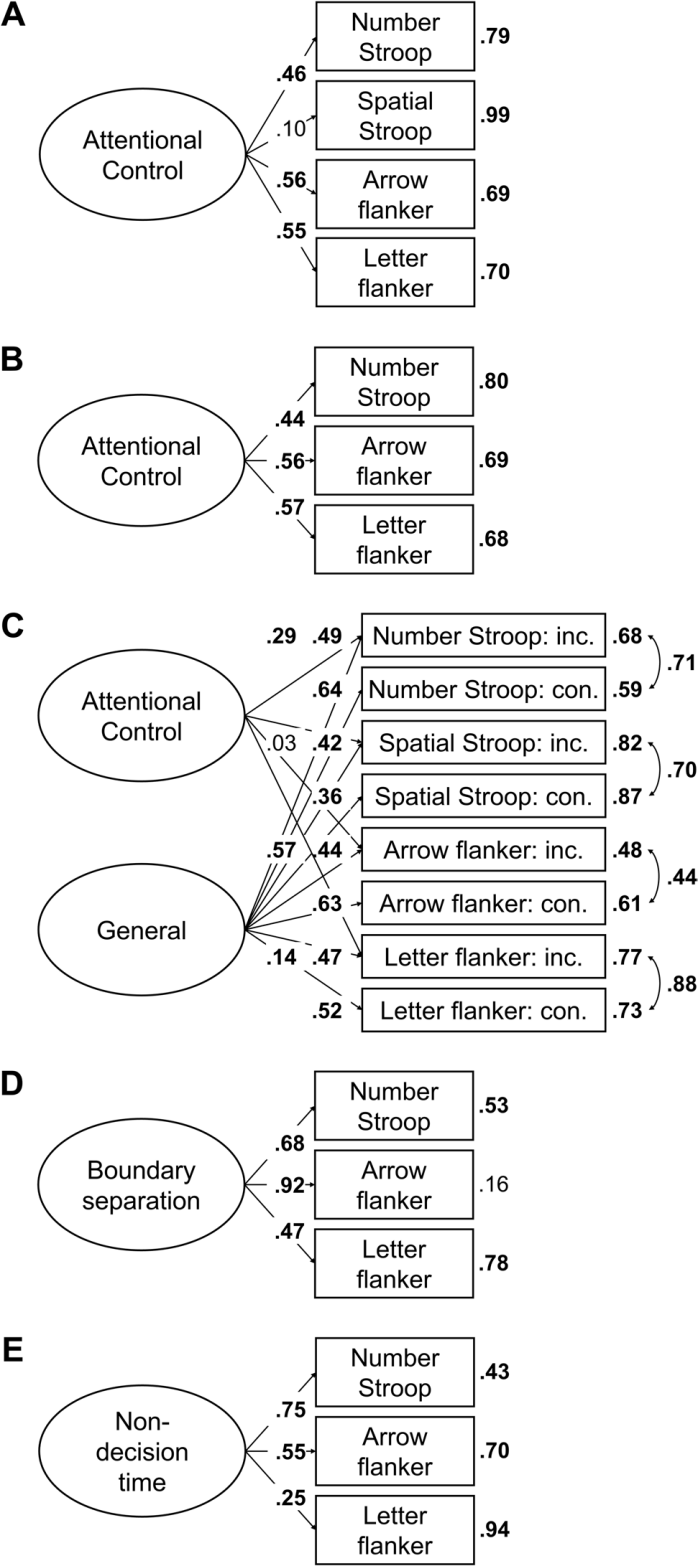
Dataset 6: Kane et al. (2016). **A** Drift rate with difference coding and all four tasks: One-factor model in which all drift-rate differences between incongruent and congruent trials loaded on a single latent variable (Model 9). **B** Drift rate with difference coding and only three tasks: One-factor model in which all drift-rate differences between incongruent and congruent trials loaded on a single latent variable (Model 10). **C** Drift rate with condition coding and all four tasks: Bifactor model in which drift rates from the incongruent and congruent trials of all tasks was forced to load on a general factor, and drift rates from the incongruent trials were forced to load on an attentional-control factor (Model 11). In this model, factor loadings were constrained to be positive, and error variances from the measures of the same task were allowed to correlate. **D** Boundary separation with only three tasks: One-factor model in which the boundary-separation parameters loaded on a single latent variable (Model 14). **E** Non-decision time with only three tasks: One-factor model in which the non-decision-time parameters loaded on a single latent variable (Model 16). See Fig. 2 note for details

The next step was to find a coherent factor of attentional control using the four attentional-control measures. To this end, we fitted a model in which the drift-rate differences from all four tasks loaded on a single factor (i.e., Model 9). However, this model provided a fit that would just be acceptable by conventional criteria for the SEM fit indices, χ^2^(2, *N* = 443) = 8.01, *p* = 0.018, CFI = 0.94, RMSEA [90% CI] = 0.08 [0.03, 0.15], SRMR = 0.04. This model is depicted in Fig. 10A. The loadings ranged from 0.10 to 0.56, and error variances were moderate (see Fig. 10A). The factor reliability was low (ω = 0.44). Thus, the results suggest a moderately coherent factor.

Because previous research has suggested a well-fitting model with a factor on which the number Stroop, arrow flanker and letter flanker tasks loaded (Rey-Mermet et al., 2018), we fitted an additional model using these three tasks only (i.e., Model 10). This model is depicted in Fig. 10B. As this model is saturated, it provided a perfect fit to the data. All loadings were larger than 0.44, but error variances were moderate (see Fig. 10b). The factor reliability was of modest size (ω = 0.52). Thus, the results suggest a moderately coherent factor.

##### Drift rate with condition coding 

Correlations between the drift rates of incongruent and congruent conditions of the same task were in the upper range (≥ 0.50) and credible. In contrast, all correlations between the different tasks were in the lower range (between 0.07 and 0.52) but still mostly credible with only two exceptions (see Table 16). Overall, the correlation matrix had a good KMO index (0.62).

In the next step, we aimed to establish a coherent factor of attentional control using the bifactor-modeling approach. Following the procedure used for the previous datasets, we fitted Model 11 as follows. The drift rates of both congruent and incongruent conditions of all four tasks loaded on a general factor. The drift rates of incongruent trials had additional loadings on an attentional-control factor. Error variances from the measures of the same task were allowed to correlate, and factor loadings were constrained to be positive. This model is depicted in Fig. 10C. This model provided a good fit to the data, χ^2^(12, *N* = 443) = 37.60, *p* < 0.001, CFI = 0.99, RMSEA [90% CI] = 0.07 [0.05, 0.10], SRMR = 0.04. All measures for the general factor were larger than 0.36. However, for the attentional-control factor, most loadings were lower than 0.30, with the loading of the arrow flanker task dominating the other loadings (see Fig. 10C). In line with these observations, the reliability for this specific factor was low (ω_h_ = 0.13). Therefore, this model estimation did not result in a coherent factor of attentional control.

Similar to the drift rate with difference coding, we fitted a bifactor model in which only the number Stroop, arrow flanker, and letter flankers were included (Model 12). This model provided a bad fit to the data, χ^2^(3, *N* = 443) = 19.32, *p* < 0.001, CFI = 0.99, RMSEA [90% CI] = 0.11 [0.07, 0.16], SRMR = 0.04.

##### Boundary separation

 All correlations were moderate (between 0.32 and 0.63) and credible (see Table 17). Furthermore, the correlation matrix had a good KMO index (0.70).

In the next step, we fitted a model in which all four tasks loaded on a single factor (i.e., Model 13). This model provided a bad fit to the data, χ^2^(2, *N* = 443) = 15.45, *p* < 0.001, CFI = 0.97, RMSEA [90% CI] = 0.12 [0.07, 0.18], SRMR = 0.03. As before, we fitted a further model in which only the number Stroop, arrow flanker, and letter flanker tasks loaded on a single factor (i.e., Model 14). This model is depicted in Fig. 10D. As this model is saturated, it provided a perfect fit. All loadings were larger than 0.47, and error variances were relatively low (see Fig. 10D). Factor reliability was quite high (ω = 0.67). Thus, these results support the view of a coherent factor reflecting individual differences in speed–accuracy trade-off or caution that generalize across tasks.

##### Non-decision time

 All correlations were low to moderate (between 0.14 and 0.41) and all credible (see Table 18). Overall, the correlation matrix had a good KMO index (0.63).

As before, we first fitted a model in which all four tasks loaded on a single factor (i.e., Model 15). This model provided a bad fit to the data, χ^2^(2, *N* = 443) = 14.555, *p* = 0.001, CFI = 0.92, RMSEA [90% CI] = 0.12 [0.07, 0.18], SRMR = 0.05. Then, we fitted the model in which only the number Stroop, arrow flanker, and letter flanker tasks loaded on a single factor (i.e., Model 16). This model is depicted in Fig. 10E. As this model is saturated, it provided a perfect fit to the data. The loadings ranged from 0.25 to 0.75 (see Fig. 10E). This resulted in a factor reliability of modest size (ω = 0.54), suggesting a moderately coherent factor for the non-decision time.

### SEM analyses on 500 covariance matrices sampled from the posterior distribution

All SEM models so far were performed on the posterior mean covariance matrices. Next, we fitted the models for all parameters (i.e., the drift rates in both difference and condition coding parametrizations, as well as the boundary separation, and the non-decision time) in all datasets (1 to 6) to 500 covariance matrices sampled from the posterior distribution. This generated a distribution of 500 SEM fits that reflects the uncertainty of the correlation estimates, and a corresponding distribution of 500 values for each statistic reflecting model fit and model adequacy, approximating their posterior distribution.

**Fig. 11:**
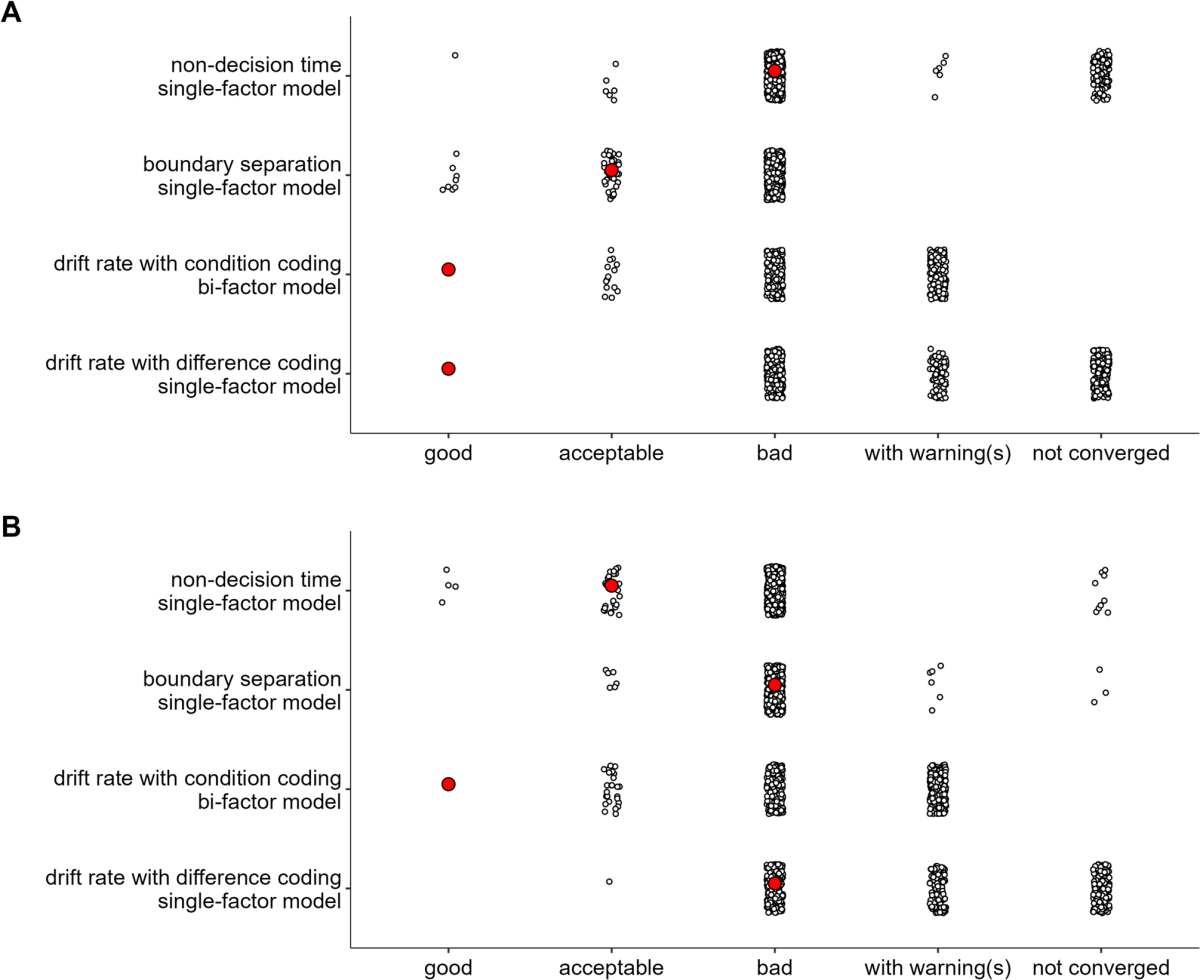
Datasets 1 and 2 from Rey-Mermet et al. (2018): Overview of the goodness-of-fit statistics for the models computed with each of the 500 covariance matrices sampled from the full posterior distribution. A model fit was considered as good if the Bentler’s comparative fit index (CFI) was larger than .95 and the standardized root mean square residual (SRMR) was smaller than .08. A model fit was considered acceptable if the CFI ranged from .90 to .95 and the SRMR was smaller than .08. Otherwise, the model fit was considered as bad. For the sake of completeness, we separately reported the cases in which the model computation resulted in some warnings (e.g., some estimated variances were negative) and the cases in which the model computation did not converge. The results for the model computations with the covariance matrix from the mean posterior distribution are presented in red. **A** Dataset 1: Young adults from Rey-Mermet et al. (2018). (B) Dataset 2: Older adults from Rey-Mermet et al. (2018). (Color figure online)

**Fig. 12:**
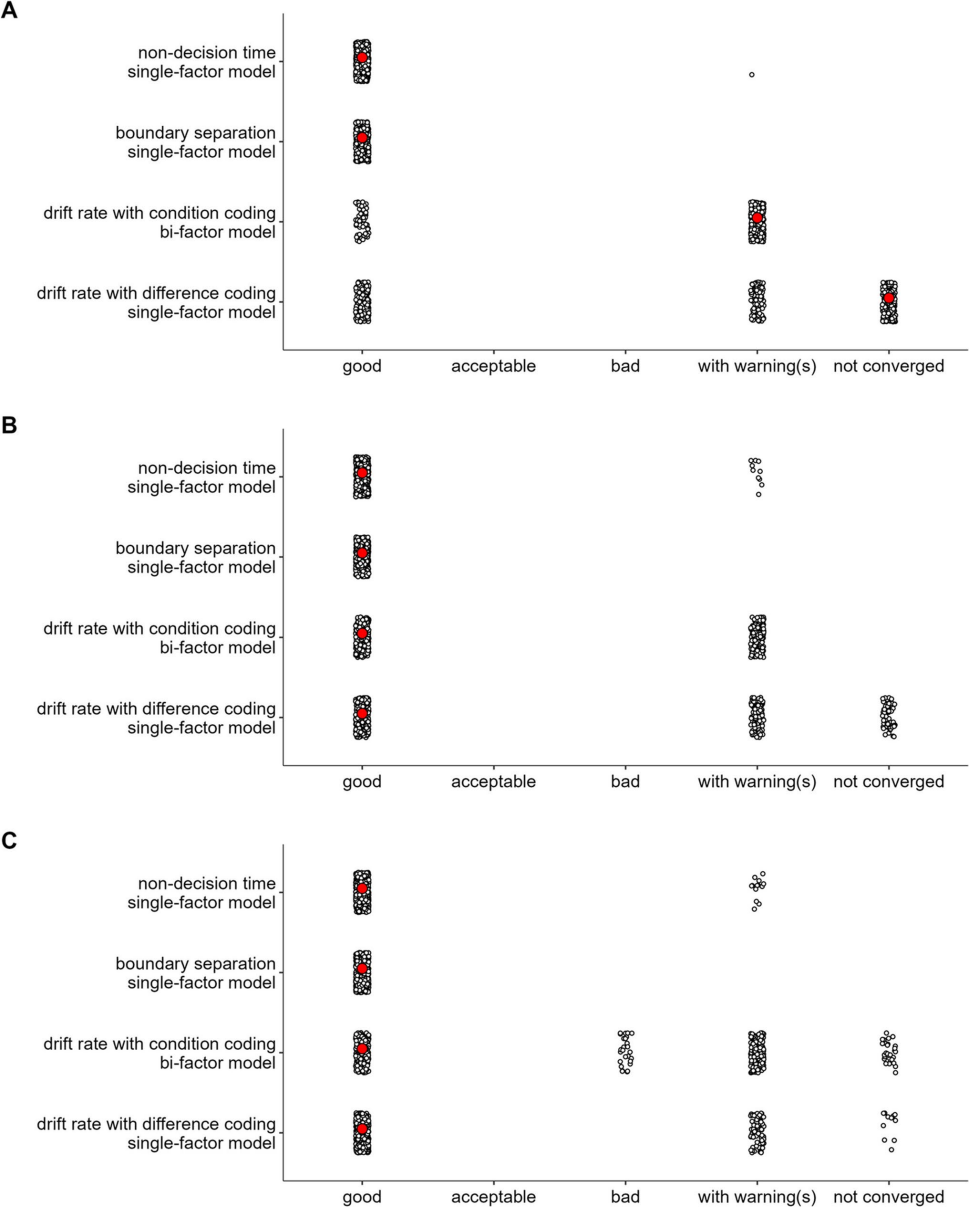
Datasets 3, 4, and 5 from Whitehead et al. (2019): Overview of the goodness-of-fit statistics for the models computed with each of the 500 covariance matrices sampled from the full posterior distribution. See Fig. 11 note for details. **A** Dataset 3: Experiment 1 from Whitehead et al. (2019). **B** Dataset 4: Experiment 2 from Whitehead et al. (2019). **C** Dataset 5: Experiment 3 from Whitehead et al. (2019). (Color figure online)

**Fig. 13:**
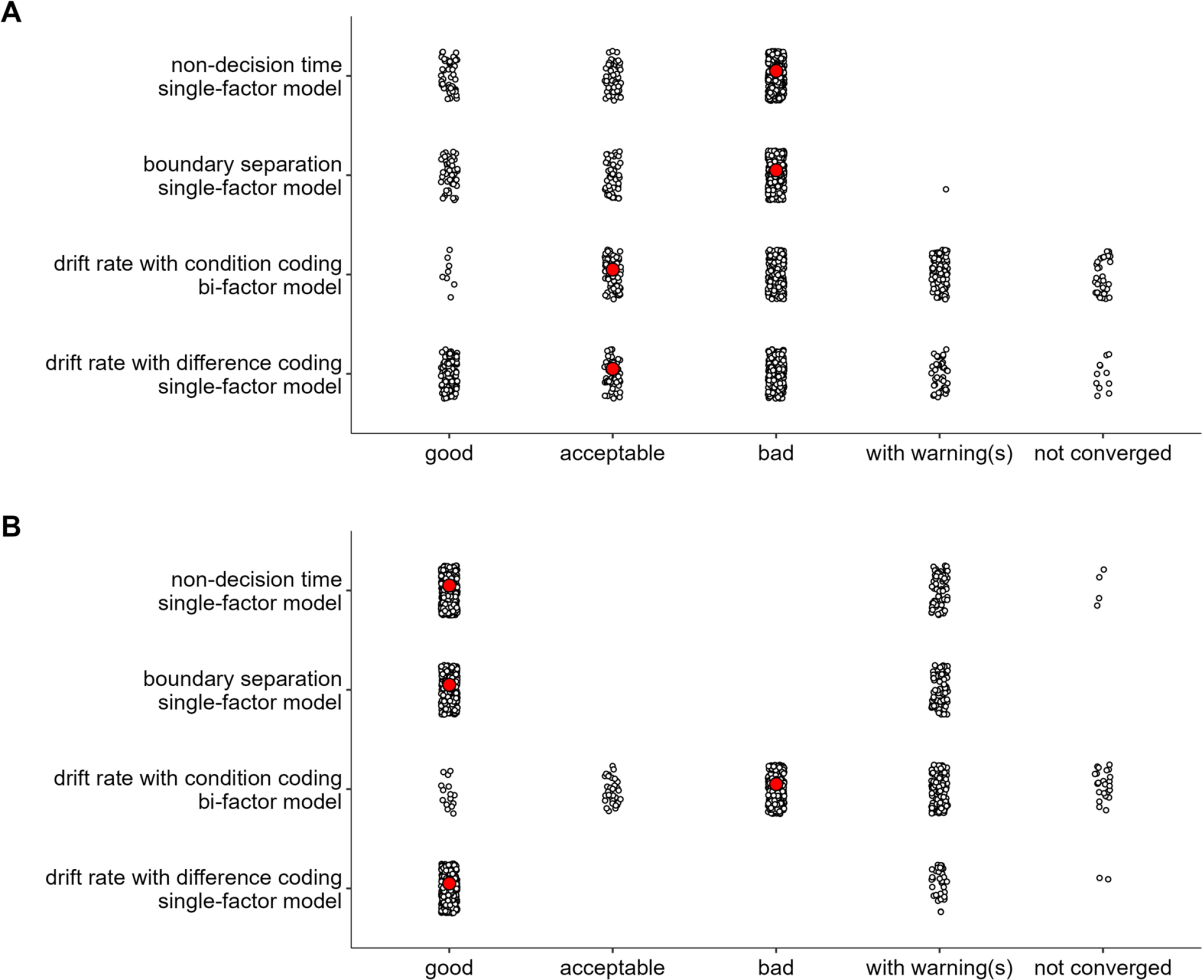
Dataset 6 from Kane et al. (2016): Overview of the goodness-of-fit statistics for the models computed with each of the 500 covariance matrices sampled from the full posterior distribution. In this dataset, the sample size consisted of 443 participants and thus was larger than 250. For this reason, the root mean square error of approximation (RMSEA) was taken into account when evaluating the model fit. Accordingly, a model fit was considered as good if the Bentler’s comparative fit index (CFI) was larger than .95, the standardized root mean square residual (SRMR) was smaller than .08, and the RMSEA values were smaller than .06. A model fit was considered as acceptable if the CFI was larger than .90, the SRMR was smaller than .08, and the RMSEA values were between .06 and .08. Otherwise, the model fit was considered as bad. See Fig. 11 note for the other details. **A** Dataset 6: Kane et al. (2016) with the models including four tasks. **B** Dataset 6: Kane et al. (2016) with the models including only three tasks (i.e., the number Stroop, arrow flanker, and letter flanker tasks). (Color figure online)

An overview of the goodness-of-fit statistics of these model assessments is presented in Fig. 11 for Datasets 1 and 2, in Fig. 12 for Datasets 3, 4 and 5, and in Fig. 13 for Dataset 6. As shown in Fig. 11, only a few models for Datasets 1 and 2 provided an acceptable fit to the data, whereas the majority of the models provided a bad fit. For Datasets 3 to 5, the single-factor models were saturated, thus provided perfect fit to the data (see Fig. 12). Nevertheless, for the single-factor models with the drift rate, there is a substantial number of cases in which the models were either only identified with warnings or did not converge at all. For the non-saturated models (i.e., the bifactor models with the drift rates), only about half of the models showed good fit statistics. For Dataset 6, when the models included the four tasks, most models provided a bad fit, were identified with warnings or did not converge at all (see Fig. 13A). In contrast, when the models included only three tasks (i.e., the number Stroop, the arrow flanker, and the letter flanker task), most models provided good fit to the data (see the bifactor model with the drift rates for an exception; Fig. 13B).

**Fig. 14:**
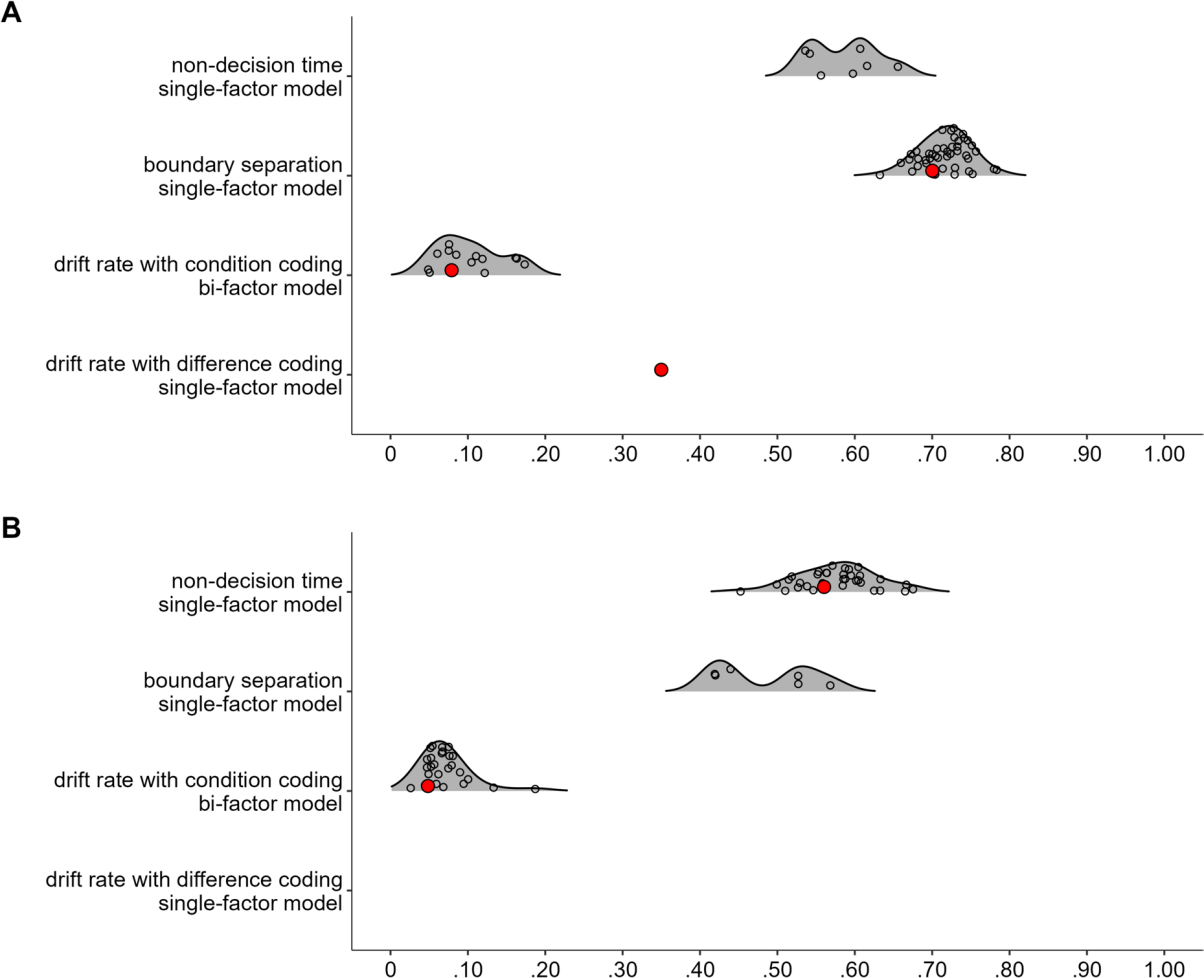
Datasets 1 and 2 from Rey-Mermet et al. (2018): Omega coefficients of factor reliability for single-factor models and hierarchical omegas for the attentional-control factor in bifactor models for which the fit statistics was acceptable to good. Fit statistics were computed with each of the 500 covariance matrices sampled from the full posterior distribution. Omegas for the model fits with the covariance matrix from the mean posterior distribution are presented in red. **A** Dataset 1: Young adults from Rey-Mermet et al. (2018). **B** Dataset 2: Older adults from Rey-Mermet et al. (2018). Please note that there is no distribution for the single-factor model in Dataset 2 because only one model provided an acceptable fit to the data (see Fig. 11b, ω = .41 for this model). (Color figure online)

**Fig. 15:**
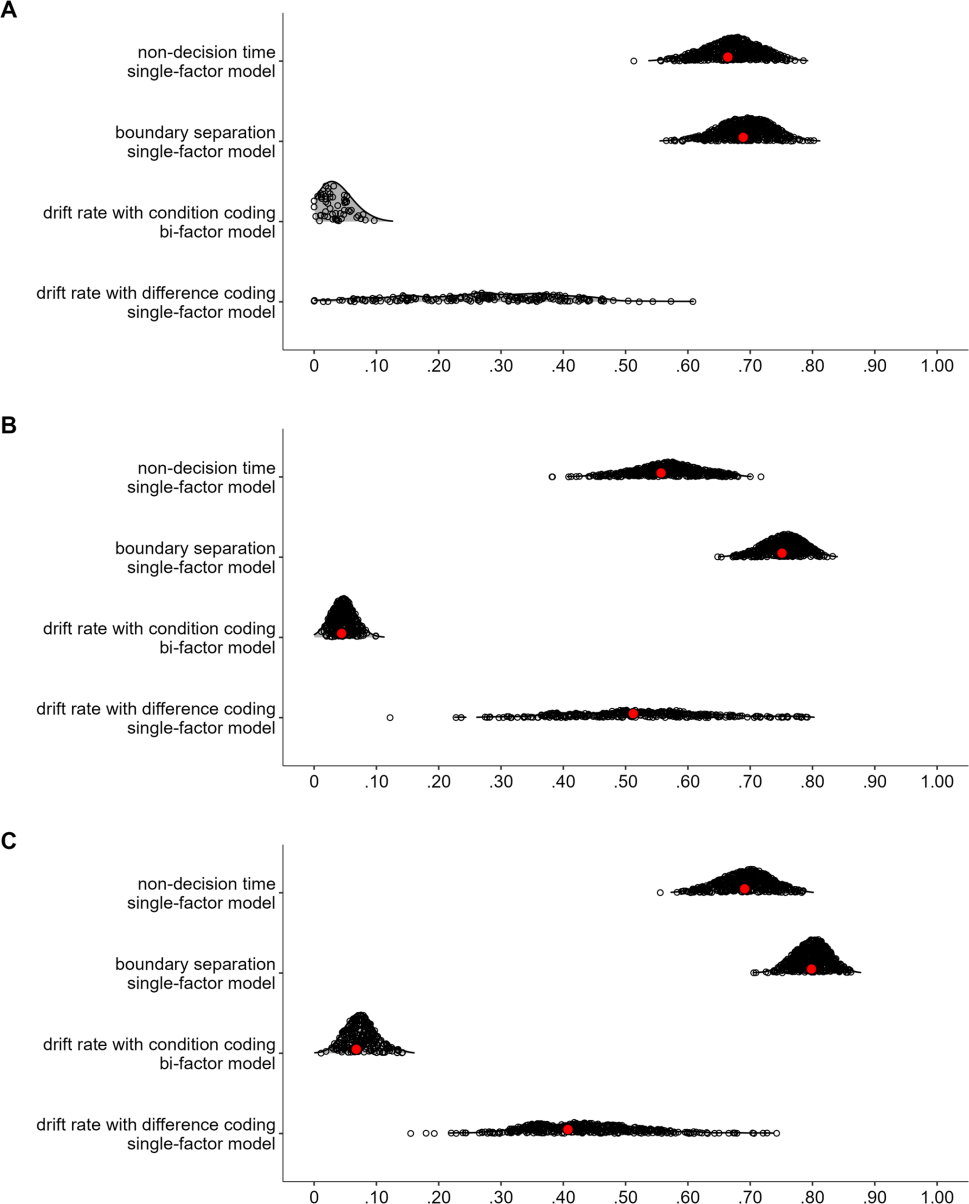
Datasets 3, 4, and 5 from Whitehead et al. (2019): Omega coefficients of factor reliability for single-factor models and hierarchical omegas for the attentional-control factor in bifactor models for which the fit statistics was acceptable to good. See Fig. 14 note for details. **A** Dataset 3: Experiment 1 from Whitehead et al. (2019). **B** Dataset 4: Experiment 2 from Whitehead et al. (2019). **C** Dataset 5: Experiment 3 from Whitehead et al. (2019). (Color figure online)

**Fig. 16:**
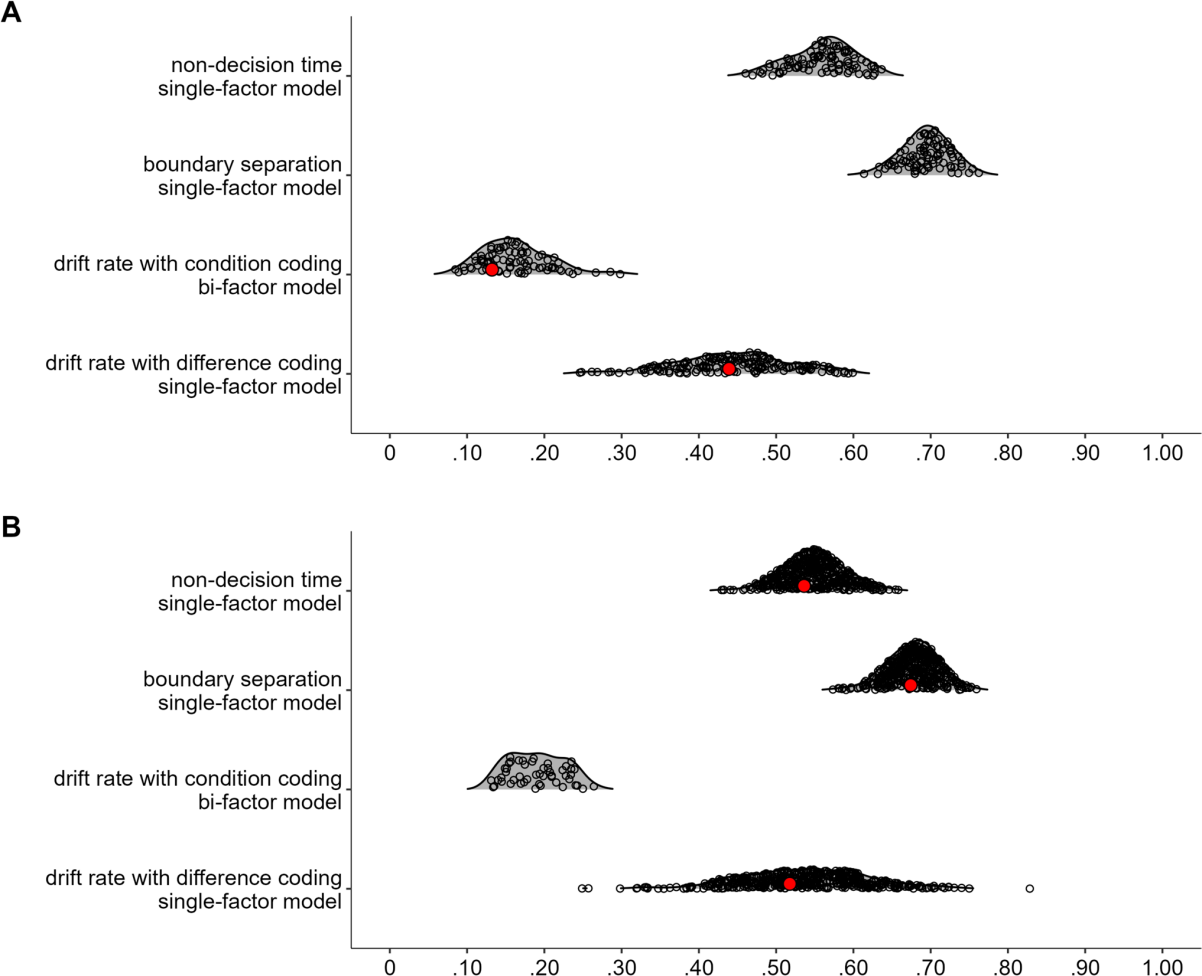
Dataset 6 from Kane et al. (2016): Omega coefficients of factor reliability for single-factor models and hierarchical omegas for the attentional-control factor in bifactor models for which the fit statistics was acceptable to good. See Fig. 14 note for details. **A** Dataset 6: Kane et al. (2016) with the models including four tasks. **B** Dataset 6: Kane et al. (2016) with the models including only three tasks (i.e., the number Stroop, arrow flanker, and letter flanker tasks). (Color figure online)

For the models with acceptable to good fit statistics, we then assessed the factor reliability. These estimates are presented in Fig. 14 for Datasets 1 and 2, in Fig. 15 for Datasets 3, 4, and 5, and in Fig. 16 for Dataset 6. Overall, the reliability of the attentional-control factor for the drift-rate parameters was generally low (≤ 0.60 for single-factor models and ≤ 0.10 for bifactor models), thus indicating that no coherent factor of attentional control was established. For Dataset 4 (see Fig. 15B) and, to a lesser extent, for Dataset 5 (see Fig. 15C) and Dataset 6 with the models including three tasks (see Fig. 16B), the range of the reliability extended beyond 0.70. However, this wide distribution reflects a high degree of uncertainty for this parameter, thus challenging the view of a coherent factor of attentional control in this case as well. For boundary separation, the factor reliability was higher, ranging from 0.60 to 0.85 for nearly all datasets (see Dataset 2 for an exception in which the factor reliability ranged from 0.40 to 0.60). Thus, except for Dataset 2, these results on the boundary separation indicate a coherent factor reflecting individual differences in speed–accuracy trade-off. For non-decision time, the factor reliability was more moderate and with a wider range, varying from 0.40 to 0.80 across the different datasets. This suggests at best a moderately coherent factor for non-decision time.

### Robustness of the results

To test the robustness of our results, we reran the analyses by applying the following modifications. First, because the hierarchical Bayesian Wiener diffusion modeling with the difference coding did not recover the data for all measures well in Datasets 1 and 2 (see Figs. 1 and 3), we estimated the models to several subsets of data in which the tasks with a poor diffusion-model fit were excluded. The excluded tasks are listed in Table 19 for each dataset separately. Second, because Datasets 1 and 2 included neutral trials as baseline as well, we also fitted bifactor models in which neutral conditions, rather than congruent conditions, were included as baseline conditions that loaded only on the general factor. Third, in Dataset 6, the single- and bifactor models were modified by modeling two attentional-control factors. That is, the number and spatial Stroop tasks loaded on a Stroop attentional-control factor. The arrow and letter flanker tasks loaded on a flanker attentional-control factor. Finally, in all datasets (1 to 6), all bifactor models were estimated with different constraints: (a) factor loadings were constrained to be positive, (b) error variances were constrained to be positive, and (c) neither factor loadings nor error variances were constrained to be positive.

**Table 19:** List of excluded tasks to test the robustness of the results from single-factor models in Datasets 1 and 2

Excluded tasks in Dataset 1	Excluded tasks in Dataset 2
Color Stroop	Negative compatibility
Global–local	Negative compatibility and letter flanker
Color Stroop and global–local	Negative compatibility and positive compatibility
Color Stroop and letter flanker	Negative compatibility, letter flanker, and positive compatibility
Color Stroop, global–local, and letter flanker	Negative compatibility, letter flanker, and number Stroop
Color Stroop, letter flanker, and negative compatibility	Negative compatibility, letter flanker, positive compatibility, and number Stroop
Color Stroop, global–local, letter flanker, and negative compatibility	Negative compatibility, letter flanker, positive compatibility, number Stroop, and Simon
Color Stroop, global–local, letter flanker, and number Stroop	Negative compatibility, letter flanker, positive compatibility, number Stroop, and arrow flanker
Color Stroop, global–local, letter flanker, negative compatibility, and number Stroop	
Color Stroop, global–local, letter flanker, negative compatibility, and positive compatibility	

The goodness-of-fit statistics of these model assessments and the parameter estimates can be found at https://osf.io/tyv9g/. None of these estimations resulted in a well-fitting model with a coherent factor of attentional control (i.e., with a high factor reliability, low error variances, and significant but non-dominant loadings for the attentional-control factor).

## Discussion

The purpose of the present study was to establish a coherent factor of attentional control when individual differences in speed–accuracy trade-offs and measurement error were accounted for. To this end, we reanalyzed the data from Rey-Mermet et al. (2018), from Whitehead et al. (2019) and from Kane et al. (2016) by computing SEM using the correlations parameters of a hierarchical Bayesian Wiener diffusion model (i.e., drift rates, or congruency effects on drift rates, boundary separation, and non-decision time). This combines a well-tested cognitive model of response selection—including speed–accuracy trade-offs—as measurement model with the current gold standard for statistical modelling of trial-by-trial noise (Rouder et al., 2023).

A summary of the results is presented in Table 20. Overall, the results showed that for all datasets, the congruency effects on drift rates hardly correlated across tasks. Moreover, irrespective of how we isolate attentional-control variance (i.e., using drift-rate differences in a single-factor model, or through a bifactor model of drift rates), SEM identified a model including a factor that generalizes across tasks in two cases only (i.e., in the drift-rate differences in Dataset 4 and in Dataset 6). In these cases, the reliability of the factor was of modest size, suggesting a moderately coherent factor of attentional control. However, the analyses on 500 covariance matrices sampled from the posterior distribution emphasized the uncertainty in the estimate of the factor reliability. Moreover, no coherent factor of attentional control was observed when the bifactor-modeling approach was used on Datasets 4 and 6. Together, this challenges the view of a coherent factor even in Datasets 4 and 6.

**Table 20:** Summary of the results

Dataset	Drift rate with difference coding	Drift rate with condition coding	Boundary separation	Non-decision time
1	No coherent factor of attentional control	No coherent factor of attentional control	**A coherent factor**	No factor
2	No factor of attentional control	No coherent factor of attentional control	No factor	**A moderately** **coherent factor**
3	No factor of attentional control	No factor of attentional control	**A coherent factor**	**A coherent factor**
4	A moderately coherent factor of attentional control but uncertain^a^	No coherent factor of attentional control	**A coherent factor**	**A moderately** **coherent factor**
5	No coherent factor of attentional control	No coherent factor of attentional control	**A coherent factor**	**A coherent factor**
6–four tasks	A moderately coherent factor of attentional control but uncertain^a^	No coherent factor of attentional control	No factor	No factor
6–three tasks	A moderately coherent factor of attentional control but uncertain^a^	No factor of attentional control	**A coherent factor**	**A moderately** **coherent factor**

For Dataset 6, the models were computed in two ways. First, all four tasks were used. Second, following previous research (Rey-Mermet et al., 2018), only three tasks—that is, the number Stroop, arrow flanker, and letter flanker tasks—were used. For the sake of clarity, results suggesting a coherent factor or a moderately coherent factor are presented in bold

^a^The results on the posterior mean covariance matrix suggest a moderately coherent factor of attentional control. However, the results on the 500 covariance matrices sampled from the posterior distribution emphasized the uncertainty of the estimate for the factor reliability, thus challenging the view of a coherent factor

### Boundary separation and non-decision time

Besides the drift rates, the results showed a coherent factor for the boundary separation in nearly all datasets (see Table 20). Only in Dataset 2, which includes the performance of older adults, and in Dataset 6, which includes the four tasks, no factor was identified. As boundary separation is typically interpreted as a measure of response caution (Wagenmakers, 2009), such as when preferring accuracy over speed, or speed over accuracy, establishing a coherent factor with that parameter suggests that participants applied the same response style across several tasks (see also Hedge et al., 2022; Weigard et al., 2021). This seems in line with the assumption of a consistent meta-control state (Hommel & Wiers, 2017), which enables participants to implement a response style and to vary it if required. Apparently, setting this response style is implemented less consistently by older adults and may not affect all tasks similarly.

The present study also yielded at least a moderately coherent factor for the non-decision time in most datasets (see Dataset 1 and Dataset 6 including four tasks for exceptions). Although this finding is in line with the results reported by Weigard et al. (2021), it was still surprising to observe a factor in this case because the non-decision time is defined as a parameter reflecting task-specific processes (Wagenmakers, 2009). One way to interpret this result may be to relate it to the finding that non-decision time has been reported to capture to the ability to predict the timing of forthcoming events (Van Den Brink et al., 2021). Critically, this ability to predict timing has been suggested to be related to attentional-control abilities (Broadway & Engle, 2011a, 2011b). Although this interpretation seems attractive, there exists, however, a more parsimonious way to interpret the coherent factor for non-decision time. Our hierarchical Bayesian approach does not consider contaminant responses (Ratcliff & Tuerlinckx, 2002). Thus, the individual differences in non-decision time may reflect individual differences in the fastest RTs. In this case, a factor in the non-decision time means that individual differences in the fastest RTs have a similar ordering across tasks. This similar ordering seems to be more or less pronounced, depending on the tasks used and the samples tested.

One may wonder why in addition to modeling separate drift rates for congruent and incongruent trials, we did not take a similar approach for the boundary separation and non-decision time. The main argument against this is again the statistical bias-variance trade-off (Yarkoni & Westfall, 2017). We want our diffusion model to be as complex as necessary to account for the main pattern in the data, but not any more complex. In line with this idea, psychometric applications of the diffusion model (e.g., van der Maas et al., 2011; Vandekerckhove, 2014) usually apply the simplest possible model variants, similar to our approach here. Nevertheless, we also attempted to estimate models in which either all three parameters, or only the drift rate and the boundary separation, were allowed to differ across congruent and incongruent trials. However, these models failed to converge and produced “divergent transitions” in the MCMC sampling process, indicating a pathological likelihood surface or, more colloquially, a mis-specified model (e.g., Betancourt, 2017). Thus, at least given the available data, and with the goal in mind to estimate the variance–covariance matrix with a non-informative prior for the correlation matrix (which we believe is one of the unique strengths of our approach), the model in which only the drift rate is allowed to vary between congruent and incongruent trials is not only the theoretically most appropriate model, but also the only computationally feasible diffusion model.

### Attentional control does not exist as a psychometric construct: Is this conclusion warranted?

Overall, the results of the present study indicate that even if we limit the impact of individual differences in speed–accuracy trade-offs and the impact of measurement error on the attentional-control measures, no coherent factor of attentional control emerges. Moreover, bypassing difference scores by applying a bifactor-modeling approach did not solve the issue (see also Rey-Mermet et al., 2018, 2019, 2020; cf. Draheim et al., 2019, 2021). These findings suggest that the difficulty of establishing attentional control as a psychometric construct is not primarily a measurement problem. Therefore, these findings challenge attentional control as a psychometric construct. The issues we are facing when establishing attentional control as a psychometric construct are no longer methodological but theoretical. In particular, we need to rethink either how we assess attentional control or how we think about attentional control as a psychometric construct.

One may argue, nevertheless, that these conclusions are not warranted. We next argue why, in our view, they are warranted.

#### Sufficient power?

A first concern may be whether, in the datasets we used, we had enough statistical power to establish attentional control at the latent-variable level or to accept the null hypothesis (i.e., to determine that attentional control at the latent-variable level has no empirical basis). Statistical power—the probability of obtaining a significant effect, given that there is a true effect—is only defined in the framework of null-hypothesis significance testing and therefore cannot be applied to our Bayesian analysis. In the present context, an approximately equivalent concept in Bayesian statistics is the precision of the posterior distributions. Like power, it depends on the amount of information in the data, that is, on the sample size and the number of trials per participant and condition: Less informative data translate into less precise parameter estimates, reflecting less certainty about their values. Unlike power, the precision of posteriors reflects the informativeness of the data not only for showing a true effect but also for providing evidence for the absence of a non-existing effect. Also, unlike power, precision does not depend on the a priori choice of an effect size.

The question is now: Were our correlation estimates precise enough? Inspection of Tables 3, 4, 5, 6, 7, 8, 9, 10, 11, 12, 13, 14, 15, 16, 17 and 18 shows that the correlation coefficients, which form the basis for the SEMs, are estimated quite precisely in most cases. More specifically, in most cases in which the correlations do not differ credibly from zero, the Bayesian 95% credible intervals, reflecting the width of the posteriors, are quite narrow around zero and generally exclude medium or large correlations as improbable. The data provide sufficient evidence that nearly all of the correlations of the drift-rate difference parameters across tasks are small at best (see Tables 3, 7, 11, and 15). Moreover, most of their posteriors had substantial mass on both sides of zero, implying that the correlations are equally likely to be positive (the predicted direction) or negative (which is incompatible with the idea of a general attentional control factor). More specifically, among all 71 drift-rate difference correlation parameters, only three had credible intervals with an upper bound above 0.50 (i.e., 0.50 twice and 0.52 once), whereas three correlations had a lower bound smaller than − 0.40 (with the smallest lower bound being − 0.48). This suggests that our correlation coefficients are precise enough to warrant our conclusions.

A complementary perspective on the precision of the correlations is to consider the strength of evidence for positive correlations among drift-rate parameters for both the difference and condition coding parametrizations. We consider two different metrics—both shown in Table 21—the probability that the average correlation is larger than a given threshold and the probability that all correlations are larger than a given threshold. The “average correlations” columns tell us something about the average precision of the correlation estimates. If a dataset was completely uninformative, we would expect a probability of 0.50 for average correlations > 0, and a probability of between 0.20 (for three tasks) and 0.05 (for eight tasks) for average correlations > 0.5.^3^ The degree of deviation from this pattern provides information regarding both location and precision of the average pairwise correlations. The “all correlations” columns tell us something about the evidence for the *positive manifold* across tasks, the assumption that all pair-wise correlations among drift rates or drift-rate differences are positive. If a dataset was completely uninformative, we expect the probability for all correlations > 0 to be only 0.16 for three tasks and only 0.04 for four tasks. These values thus allow a direct assessment of whether a given dataset can provide evidence for a coherent factor of attentional control.

**Table 21:** Posterior probabilities that the average drift-rate correlations are larger than a given threshold (left), and that all pair-wise drift-rate correlation parameters are larger than a given threshold (right)

	Average correlations						All correlations			
Dataset	> 0	> .1	> .2	> .3	> .4	> .5	> 0	> .1	> .2	> .3
	Difference coding									
1	.64	.41	.20	.07	.02	0	0	0	0	0
2	.57	.36	.17	.06	.01	0	0	0	0	0
3	.73	.40	.12	.01	0	0	.33	.04	0	0
4	.95	.82	.56	.27	.08	.01	.86	.56	.18	.02
5	.99	.91	.65	.30	.07	.01	.97	.75	.27	.02
6	.86	.66	.44	.20	.05	.01	.34	.03	0	0
6*	> .99	.96	.78	.39	.10	.01	.99	.90	.49	.07
	Condition coding									
1	.98	.89	.65	.32	.12	.07	.18	0	0	0
2	.95	.76	.44	.19	.10	.07	.01	0	0	0
3	> .99	> .99	.98	.82	.42	.22	> .99	.99	.85	.24
4	> .99	> .99	> .99	.96	.69	.30	> .99	> .99	.99	.73
5	> .99	> .99	.94	.72	.44	.24	> .99	.97	.60	.06
6	.99	.92	.70	.41	.21	.14	.86	.12	0	0
6*	> .99	.98	.85	.62	.32	.19	.99	.71	.07	0

Values in the “Average correlations” columns give the probability that the average of all pairwise drift-rate correlation parameters is larger than the threshold given in the top row (i.e., we first calculated the average correlation within each posterior sample across all drift-rate pairs and then calculated the proportion of all posterior samples for which this average correlation was larger than a given threshold). Values in the “All correlations” columns give the probability that all pairwise drift-rate correlation parameters are larger than the threshold given in the top row (i.e., for each posterior sample we checked whether all pairwise correlations are larger than a given threshold and then calculated the proportion of samples for which that was the case). For the difference coding parameterization, the calculation is only based on the drift-rate difference parameters. For the condition coding parameterization, the calculation is based on all drift-rate parameters. Dataset 6* is Dataset 6 but consisting of three tasks only (number Stroop, arrow flanker, and letter flanker). For Datasets 1 and 2, removing up to three tasks from consideration did not lead to meaningfully different results than the ones reported here

For the difference coding parameterizations, we generally find good evidence for average correlations > 0, with the only exceptions being Datasets 1 and 2. At the same time, we also find evidence that the average correlations are generally small in magnitude with no data set having more than 0.1 probability for correlations > 0.4. This suggests that the data overall is informative for the drift-rate difference correlations, but these correlations are likely positive and small. In line with this interpretation, for three of the datasets (Datasets, 4, 5, and 6 including three tasks), we additionally found strong evidence that all correlations are > 0. Even in these cases, however, the vast majority of posterior probability is concentrated on small correlations between 0 and 0.2.

For the condition coding parameterizations, the evidence for the average correlations between drift rates being > 0 is much stronger than for difference coding; it approaches 1. For Datasets 1, 2 and 6, most of the evidence points to medium sized correlations with the majority of posterior probability concentrated on values between 0.1 and 0.3 or 0.4. For the other datasets, the majority of the posterior probability is concentrated on higher average probabilities. For example, for Dataset 4, most of the posterior probability is concentrated on average correlations between 0.3 and 0.5 with even 0.3 probability for correlations > 0.5. If we consider the evidence for the positive manifold that all correlations are positive, only Datasets 1 and 2 show strong evidence against that assumption. For all other datasets, there is substantial posterior probability that all correlations are positive. For Datasets 3, 4, and 5, there is even evidence that all correlations are > 0.2. Thus, the data considered here is clearly informative with respect to the precision, location, and even direction of the pairwise correlations. Nevertheless, in the structural equation modeling approach, we failed to find evidence for a coherent factor of attentional control when using the bifactor model. Thus, the positive manifold we observed among drift rates only supports the general factor but not the specific attentional control factor.

The generally positive assessment of the precision with which we estimated the correlations contrast with the results reported by Rouder et al. (2023) who also used Datasets 1 and 2. Their results showed low correlations suffering from a large degree of imprecision (i.e., wide posterior distributions). However, even though they also used a hierarchical Bayesian model, their model assumed that response times follow a normal distribution, an assumption that the data clearly violates. Thus, it appears that our approach of using a response time distribution more appropriate for the data, the Wiener diffusion model, improves the precision of the correlation estimates to a noticeable degree.^4^ With such an approach, it is possible to draw conclusions from existing studies using attentional-control tasks. The conclusion that the data suggests is that no psychometric construct of attentional control exists.

#### What about reliability?

One may also wonder why we did not compute reliability estimates, although we were interested in the impact of measurement error. The reason is that we used a Bayesian modelling approach, which treats measurement error differently from classical test theory. In classical test theory, there is a conceptual separation between true score and error. In Bayesian parameter estimation, there is no such separation. Measurement error is instead represented in the precision of the posterior distribution. High precision of the posterior distribution indicates low measurement error, whereas low precision indicates high measurement error. Thus, in a Bayesian modelling approach, it is not appropriate to compute reliability as proposed in classical test theory to assess the measurement error. As highlighted above, the results of our approach showed that the correlations were estimated quite precisely in most cases. Therefore, we can conclude that our approach successfully reduced the impact of measurement error.

One may, nevertheless, argue that we could still compute reliability estimates by splitting the data into two halves and estimating the Bayesian model independently to each half. However, this approach is unsuitable. The reason is that this approach would only use half of the data for each estimation step, which would decrease the amount of information used to compute the parameter estimates. Because the parameter estimates of each individual participant are informed by all data of all other participants (e.g., Rouder & Haaf, 2019), this would counteract the reduction of the measurement error that should be achieved with a hierarchical Bayesian framework. Thus, the measurement error may bias the reliability estimate to an unknown degree downwards, thus making such reliability estimates less or uninformative.

Another approach to compute reliability estimates would be to estimate the reliability of a parameter from the corresponding trial-by-trial variability (Rouder et al., 2023, Footnote 1). This is in principle possible as the full diffusion model includes a parameter for the trial-by-trial variability of drift rate. However, estimating this variability parameter reliably is itself extremely difficult (Boehm et al., 2018). Thus, it is unclear whether a reliability estimate based on an unreliable variability parameter would be particularly insightful. Furthermore, to strike a good balance between bias and variance when estimating the diffusion model, we decided not to include trial-by-trial variability parameters in our model.

#### The right diffusion model?

A third concern may be whether we used the right diffusion-modeling approach. Previous research has put forward, for example, the diffusion model for conflict tasks (e.g., Ambrosi et al., 2019; Hedge et al., 2019, 2022; Ulrich et al., 2015) or its revision (Lee & Sewell, 2024). As these models were developed to explain experimental patterns associated with some attentional-control tasks, one may argue that these models are more suitable to estimate parameters. However, so far, this is by no means certain. The reason is that to explain these subtle experimental patterns, the diffusion models for conflict tasks are more complex. More complex models increase the risk of overfitting noise. When it comes to estimating parameters on the levels of individuals, simplified models can have better parameter recovery (see Boehm et al., 2018; van Ravenzwaaij & Oberauer, 2009). Because noise is idiosyncratic to each task, this may result in reduced correlations among tasks. Thus, the weak across-task correlations between parameters of the diffusion model for conflict tasks observed by Hedge et al. (2022) may be explained by the complexity of that model. The goal of the present study was to create optimal conditions for observing substantial correlations among the attentional-control tasks. For this reason, it was more appropriate to use simpler models, such as the Wiener diffusion model, which prevents the risk of overfitting noise in the data. Nevertheless, it is a question for further research to determine to what extent the diffusion model for conflict tasks or its revision are adequate to estimate correlations and parameters at the level of individuals.

#### Too much control for non-attentional-control processes?

A fourth concern may be whether our efforts to control for processes unrelated to attentional control might have removed specific attentional-control variance. In particular, using a design in which incongruent and congruent trials were intermixed within the same block and occurred with the same frequency could be considered suboptimal. The reason is that under these conditions (i.e., a mixed-block design with equal frequency of both trial types), attentional control has been suggested to affect performance on congruent trials (e.g., Heitz & Engle, 2007; Kane & Engle, 2003; Unsworth et al., 2004). This was interpreted as the result of attentional control being overused. That is, in a design in which half of the trials are incongruent, it might be parsimonious to use attentional control on all trials, including those trials that do not require attentional control (i.e., congruent trials). This argument may be valid for Datasets 3 and 5 in which incongruent and congruent trials were presented equally often. It may also apply for Dataset 4 in which incongruent trials were presented more often than congruent trials (see Whitehead et al., 2019), thus favorizing the overuse of attentional control for congruent trials. However, the argument of attentional control being overused is less plausible for Datasets 1, 2 and 6. The reason is that in these datasets, different types of trials or different ratio of incongruent versus congruent trials were used (see Table 1). For example, in Datasets 1 and 2, neutral trials were presented intermixed with incongruent and congruent trials (see the Simon task for an exception; see Rey-Mermet et al., 2018). Thus, incongruent trials—that is, trials in which attentional control is assumed to be required—were rare (i.e., 33%) in comparison with trials in which attentional control is not assumed to be required (i.e., congruent and neutral trials). Together, this indicates that the present results cannot be explained by the argument of attentional control being overused.

Similarly, one might argue that congruent trials are questionable as a baseline because they may involve a task conflict (e.g., Steinhauser & Hübner, 2009). For example, in the color Stroop task, when the word “red” is presented in the print color red, it may be unclear whether the decision to be performed is the one about the print color or the one about the word meaning (although both decisions would result in the same response). This uncertainty or task conflict may require attentional control, thus making congruent trials an inappropriate baseline. However, in the present study, no coherent factor of attentional control was established when neutral trials—that is, trials in which there is no task conflict because only response-relevant features are presented—were used as baseline in the bifactor-modeling analyses from Datasets 1 and 2. Together, these results challenge the assumption that congruent trials are not an appropriate baseline to remove non-attentional-control processes.

#### Full account for individual indifferences in speed–accuracy trade-offs and the difficulty of isolating attentional control from measurement error?

A further concern may be whether our modeling approach in which we combined hierarchical Bayesian Wiener diffusion modeling with SEM did fully account for speed–accuracy trade-offs and measurement error. We should acknowledge that no analysis, irrespective of its sophistication, can completely eliminate all the effects of speed–accuracy trade-offs and measurement error. Similarly, no analysis can faultlessly reconstruct what the data would have been without such contaminating sources of variance. For example, individual differences in speed–accuracy trade-offs are never fully accounted for by an analysis, unless the measurement model is perfect. However, no model is perfect. In this regard, the diffusion model has been the subject of much debate concerning how effectively it captures speed–accuracy trade-offs (e.g., Hedge et al., 2018a, 2018b; Rafiei & Rahnev, 2021). The reason is that instructed speed–accuracy trade-offs have been found to affect multiple parameters (e.g., boundary separation, drift rate, nondecision time). Moreover, these parameters can trade off against each other, potentially leading to unstable parameters across participants. One of the advantages of the hierarchical Bayesian framework is that it substantially limits this problem. Nevertheless, the speed–accuracy trade-offs can fluctuate to varying degrees across multiple participants.

This highlights that there is always the possibility that a more sophisticated modelling approach coupled with even more data is eventually able to reliably uncover a coherent latent variable reflecting general attention-control ability. However, given the considerable effort that has been invested in this question and the overall meager success rate (e.g., von Bastian et al., 2020), we argue that this possibility seems more theoretical than realistic.

#### Are the tasks used to measure attentional control adequate?

A final concern may regard the tasks used to measure attentional control. In line with previous research (e.g., Faßbender et al., 2023; Gärtner & Strobel, 2021; Löffler et al., 2024; Rey-Mermet et al., 2018, 2019, 2020), the results of the present study showed that when attentional control is measured with the tasks used most often for that purpose, like the Stroop task, attentional control cannot be established as a psychometric construct. This emphasizes that the typical tasks are not doing the job that we want them to do for the measurement of individual differences. As pointed out by Hedges et al. (2018b), in most situations, the typical attentional-control tasks do not produce sufficient individual variation in attentional control relative to measurement error (see also Rouder et al., 2023). Advanced individual-differences analyses, such as the analyses reported in the present study, can reduce the impact of this problem. It is the reason why we observed quite precise correlation coefficients. However, we should acknowledge that the present approach cannot completely eliminate this problem if the tasks do not produce sufficient individual variation in attentional control.

The difficulty of the attentional-control tasks in producing sufficient individual variation in attentional control has motivated researchers to seek alternative ways for measuring attentional control. For example, Rey-Mermet and Rothen (2025) have tested the replicability and robustness of the early structural equation models in which attentional control was extracted at the latent-variable level using working-memory tasks and short-term memory tasks (Engle et al., 1999; Kane et al., 2004). These tasks are used to assess the temporary maintenance of information and the temporary maintenance and manipulation of information, respectively. Critically, these tasks have been assumed to require attentional control (Engle et al., 1999; Miyake et al., 2000; see also Baddeley, 1996). Rey-Mermet and Rothen (2025) showed, however, that the models used to extract attentional control were not replicable. These results challenge the idea of using these models as an alternative to estimate attentional control at the latent-variable level. Furthermore, these results question the common assumption that attentional control is involved in working-memory tasks.

Other researchers have developed new tasks to measure attentional control as a psychometric construct (e.g., Burgoyne et al., 2023; Draheim et al., 2021, 2024; Martin et al., 2021). The validity of these new tasks has been assessed using zero-order and/or latent correlations between the new tasks and the typical tasks, as well as tasks used to assess other constructs, such as working memory, processing speed, fluid intelligence (i.e., the ability to reason with novel information). However, this approach is problematic for two reasons (see also Rey-Mermet & Rothen, 2025). First, using the typical measures of attentional control as a criterion is questionable because the results of the present study as well as those of previous research showed that these measures do not assess the general attentional-control ability (e.g., Faßbender et al., 2023; Gärtner & Strobel, 2021; Hedge et al., 2018a, 2018b; Löffler et al., 2024; Rey-Mermet et al., 2018, 2019, 2020; Rouder et al., 2023). Second, the correlations observed between the new tasks and working-memory, fluid-intelligence, or processing-speed tasks may be driven by other processes than attentional control. The reason is that it has not been clarified for the new tasks how attentional control was isolated from other processes. Together, this emphasizes the necessity of being cautious when using these new tasks because we do not know what we are measuring.

### Conclusion

In sum, the results of the present study show that no factor of attentional control could be established even if we limit the impact of individual differences in speed–accuracy trade-offs and the impact of measurement error on the attentional-control measures. The present study is, thus, the first to address an influential critique of difference scores (Draheim et al., 2021) by showing that using improved data-analytic methods that circumvent the limitations of difference scores do not lead to more optimistic conclusions concerning the existence of a general attention-control factor. This highlights that the difficulty of establishing attentional control as a psychometric construct is not primarily a measurement problem. Taken together, the available evidence challenges the existence of attentional control as a psychometric construct. This calls into question the widely held assumption that people differ in a general ability to control attention. It also questions research in which a general attentional-control ability is used as a construct explaining group differences, as for example, in developmental psychology, aging research, bilingualism, and psychopathology.

## Data Availability

The datasets from Rey-Mermet et al. (2018) can be accessed at https://osf.io/rygex/. The datasets from Whitehead et al. (2019) can be accessed at https://osf.io/t9c6z/. The dataset from Kane et al. (2016) can be accessed at https://osf.io/jv6yu/. Our complete analysis codes and results are available at the Open Science Framework (OSF) and can be accessed at https://osf.io/tyv9g/.

## Footnotes

1 In their study, Yangüez et al. (2024) concluded that when attentional control was measured using only the drift rates of the high attentional-control conditions, their results provided evidence for a factor of attentional control. However, this conclusion is questionable for two reasons. First, Löffler et al. (2024) have shown that when only the drift rates of the high attentional-control conditions are used to estimate a factor of attentional control, there is no variance left that can be explained by attentional control. All the variances could be explained by processing speed. Second, Yangüez et al. (2024) focused on model comparison and convergence rates. No information was provided about whether the models with the best fitting and high convergence rates included coherent factors.

2 In Whitehead et al. (2019), this task is designated as a Simon task. To distinguish this task from the Simon task used by Rey-Mermet et al. (2018) and to be consistent with most previous research (e.g., Hedge et al., 2022; Kane et al., 2016), we referred to this task as a spatial Stroop task.

3 These values were obtained via simulation; see R code here: https://osf.io/h9qu4

4 Rouder et al. (2023) also report (their Footnote 2) that in their simulations, the inverse-Wishart prior for the variance–covariance matrix showed “better performance” than an approach using an LKJ prior. They attribute this to the ability of the inverse-Wishart prior to constrain the scale of individual differences on the level of the response times. In our approach, the diffusion model already constrains the scale of the data by explicitly modelling the right-skewed shape of response time distribution. Furthermore, individual differences are not modelled on the level of the response times but on the level of the diffusion model parameters, for which prior information about the scale is not readily available. Thus, given the general preference for the LKJ prior in the literature (e.g., McElreath, 2020), it seems unlikely that the same would hold here.

## Declarations

### Open Practices Statements: Availability of data and materials & code availability

For the reanalysis of all datasets, we report all data exclusions, all manipulations, and all measures. The data we used are available at https://osf.io/rygex/ for the datasets from Rey-Mermet et al. (2018), at https://osf.io/t9c6z/ for the datasets from Whitehead et al. (2019), and at https://osf.io/jv6yu/ for the dataset from Kane et al. (2016). All analysis codes and results are available at the Open Science Framework (OSF) and can be accessed at https://osf.io/tyv9g/. The data analyses were not preregistered.
